# Kin Recognition in a Clonal Fish, *Poecilia formosa*

**DOI:** 10.1371/journal.pone.0158442

**Published:** 2016-08-02

**Authors:** Amber M. Makowicz, Ralph Tiedemann, Rachel N. Steele, Ingo Schlupp

**Affiliations:** 1 Department of Biology, Ecology and Evolutionary Biology, University of Oklahoma, 730 Van Vleet Oval, Norman, OK 73019, United States of America; 2 Department of Biology, Lehrstuhl für Zoologie und Evolutionsbiologie, University Konstanz, Universitätsstraβe 10, 78457 Konstanz, Germany; 3 Unit of Evolutionary Biology/Systematic Zoology, Institute of Biochemistry and Biology, University of Potsdam, Karl-Liebknecht-Strasse 24–25, 14476 Golm, Germany; Universita degli Studi di Padova, ITALY

## Abstract

Relatedness strongly influences social behaviors in a wide variety of species. For most species, the highest typical degree of relatedness is between full siblings with 50% shared genes. However, this is poorly understood in species with unusually high relatedness between individuals: clonal organisms. Although there has been some investigation into clonal invertebrates and yeast, nothing is known about kin selection in clonal vertebrates. We show that a clonal fish, the Amazon molly (*Poecilia formosa*), can distinguish between different clonal lineages, associating with genetically identical, sister clones, and use multiple sensory modalities. Also, they scale their aggressive behaviors according to the relatedness to other females: they are more aggressive to non-related clones. Our results demonstrate that even in species with very small genetic differences between individuals, kin recognition can be adaptive. Their discriminatory abilities and regulation of costly behaviors provides a powerful example of natural selection in species with limited genetic diversity.

## Introduction

Kin selection theory predicts that cooperative and altruistic behaviors scale with relatedness [1–5], strongly favoring close relatives. This has been shown empirically in numerous sexual species (Insects [6–7]; Frogs [3–4]; Fish [8–10]; Birds [11]; Mammals [5]). But how large must the difference in relatedness be for kin recognition to occur [3]? To address this, we need to understand just how relatedness shapes social behavior in species with the highest possible relatedness between individuals: clonal organisms. Like monozygotic twins in humans, clonal organisms are genetically extremely similar, sometimes completely identical. Young human twins are almost impossible to tell apart by the naïve observer, but with some experience there are often subtle differences that allow us to distinguish between individuals [12]. While some clonal invertebrates are capable of detecting and favoring full clonal sisters, others lack the ability to discriminate between their own and other clonal lineages [13–18]. Indeed, it would seem that a major form of selection, kin selection, is eliminated because the genetic variation that allows for discrimination is so minute that discrimination becomes unlikely, or the cost of altruistic behaviors become too high (i.e. limited dispersal, increased competition among relatives) [19]. These findings raise three important questions: how much genetic variation is required for kin recognition to evolve, is recognition eliminated because there are no available mechanisms in clonal organisms, and what is the adaptive benefit of kin recognition among clones? We investigated these questions using an ameiotic, clonal fish, the Amazon molly (*Poecilia formosa*), which naturally occurs in mixed groups of different clones [20–21].

The Amazon molly is a natural hybrid species that reproduces via sperm-dependent parthenogenesis, or gynogenesis, and originated from a hybridization event between *P*. *latipinna* and *P*. *mexicana* approx. 120,000 generations ago and has evolved into a species with extremely limited within-species variability [20,22]. The diploid eggs of *P*. *formosa* are pseudo-fertilized by either male *P*. *latipinna* in south Texas or *P*. *mexicana* in east Mexico, and typically the male genome is not incorporated into the offspring, leading to identical daughter clones [21,23]. Mollies are livebearing and have internal fertilization, and sexual and asexual females compete for the same males [24]. Gynogenesis results in populations of Amazon mollies that are genetically relatively uniform, yet clonal lineages may occasionally diversify by introgression, mutation, or gene conversion [21], and several different clonal lineages are known to coexist within the same population [20–21].

Amazon mollies show great similarities with their sexual hosts in their ecological niche, including feeding behavior, mating preferences, parasite loads, life history traits, fecundity, and survivorship [25–29]. They live in very fluid social environments with their host species, which change constantly in the composition of sex, species, and even clone lineage [25]. This competition should favor targeted aggressive behaviors, which in turn should favor species and potentially kin recognition. In the present study, we test the ability of *P*. *formosa* to distinguish clonal sisters (i.e., females of the same clone born of the same mother) from non-sisters. We further establish the sensory systems used in this recognition, and provide an adaptive explanation for the evolution of kin recognition by testing if aggressive behaviors scale directly with relatedness.

Given the high genetic similarity with other clonal lineages, competition for resources, low dispersal rates of the clones, the high diversity of clonal lineages with in a population, and the social environment in which they occur, we hypothesize that Amazon mollies show the ability to detect different clonal lineages and adjust their aggressive behaviors accordingly.

To test this hypothesis, we created six clonal lineages by mating virgin Amazon mollies from populations collected from the entire geographical range of the species to sailfin molly males (S1 Fig, S1 Table). Clonality of each lineage was confirmed using microsatellites (S2, S3 and S4 Tables). The results indicate that our clonal lineages exhibit: 1) high degrees of relatedness within each clonal lineage of or close to the value of 1; and 2) lower relatedness between clonal lineages in comparison (S5 and S6 Tables). We define clonal sisters as those individuals that are genetically identical, based on microsatellites, to the focal females and are descendants of the same founding mother. Non-sister individuals are defined as females that originate from a different, more distant clonal lineage and are not genetically identical to the focal females (i.e., as related to the focal females as random, non-kin individuals of a sexual species). Additionally, Amazon mollies show considerable individual variation in behaviors (i.e., preferences, aggression, etc.) [24,30–31] within and among clonal lineages, suggesting that after establishing kin recognition in multiple lineages, the use of a single lineage to further explore kin recognition within this species is sufficient.

## Material and Methods

### Populations

A single female each from six populations across the geographic range of *Poecilia formosa* (S1 Fig) was isolated and kept with a male *P*. *latipinna* (Comal Spring, TX) to found the clonal lineages (S1 Table). Populations were maintained in outdoor tanks (1000L) during the summer and indoor tanks in the winter and fed tropical fish flakes *ad libitum*. After several generations (4±2 generations), tissue samples were collected to confirm that the population was a single clonal lineage. We used 12 microsatellites to analyze the genetic divergence between the different populations (S2 Table) [32]. We then compared loci, H_0_, H_E_ of each of the different clonal lineages. We also assessed divergence among lineages, by calculating the F_ST_ values among lineages, both locus-wise and across all loci, and by performing exact tests of differentiation using Markov Chain Monte Carlo simulations (S2 and S3 Tables) [33]. Genotypes of females indicate that each female within the clonal lineages is indeed identical to one another. We calculated the genetic identity and relatedness coefficient within and among the clonal lineages (S5 and S6 Tables) [34–35]. We found that although all Amazons are closely related, females clustered together based on clonal lineage. We also wanted to investigate kin recognition within a population, and isolated two different clones from Comal Spring, TX. These clones only differed at two microsatellite loci (GA-V18: 122–144 vs. 122–148; GT-II33: 182–182 (homozygous) vs. 178–182). Together this allows us to address the minimum genetic distance required for kin recognition to occur within a population and between populations. Note that one clonal lineage, Comal Spring 7a, was genetically indistinguishable from that of San Ignacio with the 12 microsatellites that we tested for (S4 Table**)**.

### Kin recognition

**Fig 1:**
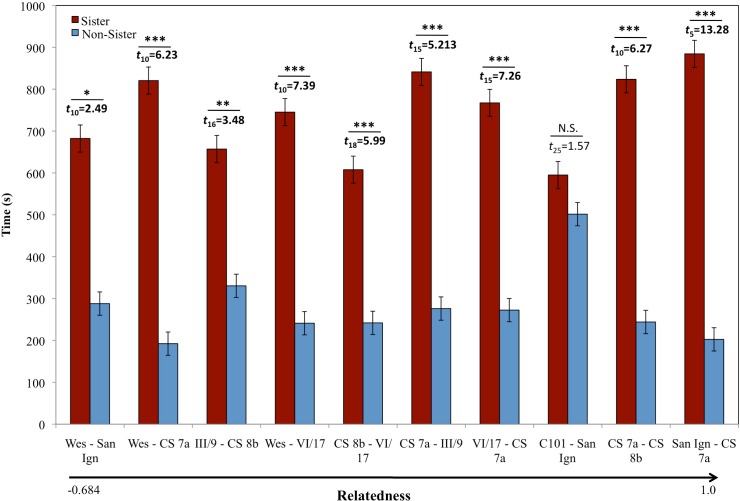
Female Kin Preference. The average time ± SE female preferences for clonal sisters (red) and non-sisters (blue) in six different clonal lineages across the range of P. formosa. Relatedness between the focal females and the nonsister clones scaled from left (more distant) to right (identical). Weslaco (Wes) paired with non-sister San Ignacio (San Ign); Weslaco paired with non-sister Comal Spring 7a (CS7a); III/9 Barretal (III/9) paired with non-sister Comal Spring 8b (CS8b); Weslaco paired with non-sister VI/17 Nuevo Padilla, (VI/17); Comal Spring 8b paired with non-sister VI/17 Nuevo Padilla; Comal Spring 7a paired with non-sister III/9 Barretal; VI/17 Nuevo Padilla paired with non-sister Comal Spring 7a; County 101 San Marcos (C101) paired with non-sister San Ignacio; Comal Spring 7a paired with non-sister Comal Spring 8b; and San Ignacio paired with non-sister Comal Spring 7a. Females from 6 of the 7 populations showed a significant preference (* = *p*<0.05; ** = *p*<0.009; *** = *p*<0.0001; NS = non-significant) for clonal sisters over non-sisters when visual, chemical and mechanical information was present. For unknown reasons, C101 clonal lineage had relatively low genetic identity, likely leading to a lack of kin recognition in this population.

**Fig 2:**
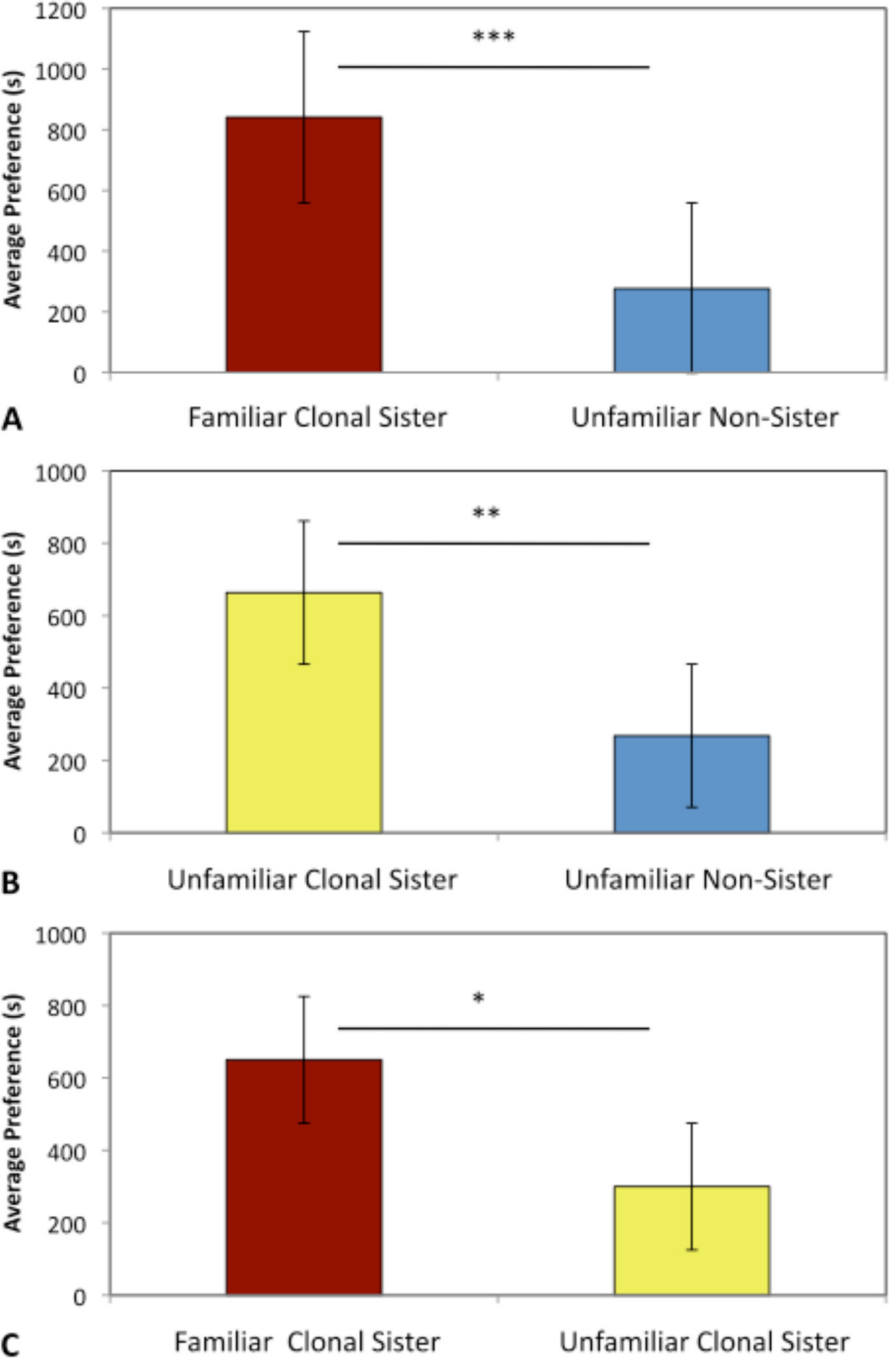
Preferences for Familiarity. The average ± SE for female preferences of clonal sisters and non-sisters was not due to familiarity. In each experiment, 15 females were given a choice between a familiar clonal sister (red), an unfamiliar clonal sister (yellow), or an unfamiliar non-sister (blue) after a 10-month isolation period from clonal sisters. **A.** Females from Comal Spring, TX, familiar clonal sisters and unfamiliar non-sisters, *t*_(15)_ = 5.213, *p*<0.0001; **B.** Unfamiliar clonal sisters and unfamiliar non-sisters, *t*_(15)_ = 3.362, *p* = 0.005; **C.** Familiar clonal sisters and unfamiliar clonal sisters, *t*_(14)_ = 2.966, *p* = 0.011. Females maintain the preference for clonal sisters (regardless of familiarity) and prefer familiar clonal sisters to unfamiliar clonal sisters.

A standard binary choice test (S3 Fig) [36–37] allowed *P*. *formosa* to choose between clonal sisters and non-sisters (a clone from another population). The stimulus fish (size matched females, ±3mm) were placed in clear, perforated Plexiglas cylinders (to allow chemical, visual, and mechanical cues) at each end of the experimental tank (61x39x30cm; Note: these Plexiglas cylinders differed depending on the protocol to test specific types of cues). The focal females were then placed into the center of the tank inside a Plexiglas cylinder to permit chemical, visual, and mechanical cues to reach them and were then allowed to acclimate for 10 minutes. After this period, the association time (s) females spent in the preference zone with a stimulus female was recorded. The experiment did not begin until the female began swimming freely. To prevent any side bias, focal females were tested twice, with the second trial having the partner females switching sides (with exception to the mechanisms experiment, see below) [37]. These two trials were added together and the strength of preference (SOP) scores were calculated as: The total time spent with Stimulus 1 / (Total time spent Stimulus 1 + Total time spent Stimulus 2). These SOP scores were calculated for both the clonal sisters and non-sisters, then √arc(sin) transformed to normalize the data. Paired *t*-tests were used to compare the transformed SOP (Strength of Preference) scores for clonal sisters and non-sisters in SPSS (ver. 17, Figs 1 and 2).

### Mechanism of kin recognition

Focal females and clonal sisters came from a stock population originally collected from the Río Purificacíon in Nuevo Padilla (VI/17), Mexico, and non-sister stimulus females originated from Comal Springs, Texas. Fish were maintained in a 12:12 light:dark photoperiod, and fed fish flakes *ad libitum* daily. Focal females were randomly selected from the stock populations and then isolated from clonal sisters for a minimum of one-week prior to conducting the experiments in a separate 75.7L tank. Stimulus female populations were maintained under similar conditions in separate 37.9L tanks.

To prevent effects from residual chemical signals on female preferences, prior to each individual experiment, the experimental tanks, Plexiglas cylinders and Plexiglas sideboards were washed with soapy water, and then rinsed thoroughly, followed by 3% hydrogen peroxide [38]. After the tanks were clean, the experimental tank was filled with ionized water (700–1000ppm). White Plexiglas was placed on the bottom and both long sides of the tank to prevent any influence by the presence of the experimenter. Treatment Plexiglas cylinders were randomly assigned and placed in each end on the tank.

Experimental treatments consisted of: 1) allowing visual only cues of the stimulus females to be passed to the focal fish using solid, clear Plexiglas cylinders; 2) visual and chemical cues using clear cylinders perforated with small 3mm holes; 3) chemical only cues using solid black cylinders perforated with small 3mm holes (corresponding to an area of about 7mm^2^ per hole, to reduce neuromast stimulation); 4) only chemical and mechanical (lateral line) signals using black cylinders with large 5mm holes (equivalent to about 20mm^2^ per hole, to increase neuromast stimulation); 5) a side bias control using clear empty cylinders; and 6) a color control to compare the response effects of one empty black and a clear cylinder. The area perforated was the same for cylinders with small or large holes (see S4 Fig for visual of experimental cylinders).

A standard binary choice test was used as described above. Individual trials were run for 10-minutes using the Viewer video tracking system (BIOBSERVE GmbH, Bonn, Germany) [39] to record the time spent near each stimulus and the number of times the focal fish entered each preference zone. At the conclusion of the trial, each female was placed into an individual tank (3.8L) overnight, which allowed us to identify each focal and stimulus female. On the next day, the same procedures were employed until all six treatments were completed. The order in which the treatments were presented and the side each stimulus female was placed was randomized. During the entire duration of the experiments females were maintained in similar lighting conditions as above, and fed frozen mosquito larvae.

SOP scores were calculated using the time spent within the preference zone, and then √arc(sin) transformed to normalize the data. A repeated-measures GLM was run using “treatments” as the within-subject variable. To analyze differences between unimodal (e.g. visual only) and bimodal (e.g. visual plus chemical) sensory mechanisms a repeated-measures GLM was used using “mode” as the within subject factor.

Prior to experimentation, we validated the construction of the Plexiglas cylinders so that they were only allowed the specific signals for each type of cylinder to be released. To demonstrate the diffusion of the chemical cues from the inside of the Plexiglas cylinders we ran several trials using food coloring. Using an identical set up as previously mentioned, we placed a fish inside the cylinder and then added 10 drops of red food coloring (Ingredients: water, propylene glycol, FD&C red, and propylparaben; Note: this was not harmful to the fish) and measured the time it took to diffuse to the preference zone. We ran this trial for all four types of Plexiglas cylinders to confirm their construction (i.e., to confirm that the solid cylinder was indeed only allowing the visual only signals and did not leak chemical signals; S4 Fig).

### Kin recognition in natural water

We wanted to determine if clonal recognition was an artefact of the laboratory or if it indeed occurs in natural habitats. To do this, we tested a population of Amazon mollies (Weslaco, Texas), which have already displayed clonal recognition in the laboratory, to determine if they can recognize sister clones under natural water conditions. In addition, we wanted to test if Amazon mollies can use visual only signals to recognize clonal sisters from non-sisters, or if turbidity, which is high in many of the Amazon mollies’ natural habitats, would make chemical signals more dominant when visual accuracy is limited.

The non-sister clone population used was a population that originated from Mexico. In practice, we collected fishes from a site in Weslaco, Texas, separated them into two aerated containers (one for focal females, the other for clonal sisters; 45.4L), and transported them to a local hotel. They were allowed to settle in over night and were then used in the experiment described below. Water (76L) from the field site that included native chemical and visual “noise” (i.e., pheromones and turbidity), was also brought into the hotel to allow us to test if the recognition signals are still detectable in their natural environment. We used this water in the subsequent tests. We had to dilute the water due to naturally high turbidity (day of collection: 246 NTU; average: 242.3±120.1; maximum: 482.5; minimum: 82.9); therefore, we added 19L of spring water to 38L of native water. Although diluting the water would likely have influenced both chemical and visual signals of the natural water source, our results suggest that even with this diluted water the visual signals were still impacted by the turbidity. Due to naturally high turbidity levels of this environment and the inability of identifying different clones visually, a field study within the natural water source was impossible; therefore, we were forced to use this experimental design in the field. Indeed, it would be unethical to introduce the non-sisters from Mexico into an open field design, thus we designed the above protocol to best address this question. Tanks and other equipment used, was identical to those used in the laboratory experiments described above. All fish were kept in aerated 45.4 L containers.

We performed 2 different experiments: 1) testing chemical only signals using black Plexiglas cylinders, perforated with 3 mm holes, and 2) testing visual only signals with solid clear Plexiglas cylinders. The sister individual was a wild caught female from the field site in Weslaco, Texas, while the non-sister individual was from a population in Mexico (see above). A standard binary choice test (S3 Fig) was used. At the conclusion of the first experiment, females were then tested in the second experiment using the same procedure. The SOP scores were calculated and a repeated measures GLM was used to compare the two different experiments, and *t*-tests were used as post-hoc analyses to compare with-in the experiment.

### Diet influence on chemical recognition

Fish originated from two of the single clonal lineages above (San Ignacio and Weslaco). Pregnant females were collected from stock tanks and isolated in individual tanks (3.8L) until they had offspring. Adult females were then returned to the stock tanks. Broods were raised together for a total of five weeks in 12/12 hour light/dark cycle and fed *ad libitum* brine shrimp and flake food. At 5 weeks of age, juveniles were raised individually in 3.8L tanks on either: 1) a high protein (crude protein 52% min.) diet, 2) a low protein (crude protein 37% min.) diet, or 3) a 50/50 mix of the high protein and low protein (crude protein 44.5% min.) diet. Visual communication between the tanks was prevented to avoid visual imprinting from the neighboring tanks as the juveniles grew. Each tank had weekly 2/3 water changes. The tank temperature was maintained at 27.8°C during the duration of the experiment. Individuals were raised until 22–34 weeks old prior to the start of the behavioral experiments.

To measure preferences, a standard binary choice test was used. However, stimulus females were placed into black perforated Plexiglas rectangular cylinders on either end of the experimental tank (18.9L). These black cylinders allowed focal females to make their choice solely based on chemical cues. After the experiment was finished the focal and stimulus females were returned to their appropriate individual tanks. We used five, randomized treatments to assess whether females would retain clonal recognition when females were placed on different diets: 1) a clonal sister on a different diet vs. non-sister on same diet; 2) a clonal sister on different diet vs. non-sister on mix diet; 3) a clonal sister on mix diet vs. non-sister on same diet; 4) a clonal non-sister on same diet vs. non-sister on different diet; and 5) a clonal non-sister on same diet vs. non-sister on mix diet. Focal females were retested every 24-hours until they complete all five treatments. If a female did not respond within the first 5 minutes of the trial, the trial was terminated, and the female was returned into her appropriate tank and retested the next day. Females that were used as stimulus females were not tested as focal females until one week had passed. Females that were focal females were used as stimulus females only after all five treatments were complete.

Shoaling preference was analyzed using a preference function test (S5 Fig). Using a block design, we randomly tested half of the females as focal females and used the other half to compose the stimulus shoals; after one week, the females were switched and the second half of the females were tested as focal female with the first half as was used as stimulus females. We used five randomized treatments, and one treatment was tested every 24 hours: 1) a clonal sister shoal on the same diet, 2) a clonal sister shoal on a different diet, 3) a non-sister clonal shoal on the same diet, 4) a non-sister clonal shoal on a different diet, and 5) a control where the black Plexiglas cylinder was present in the test tank but empty. Focal females were placed in a perforated Plexiglas cylinder on the side of the tank opposite the shoal Plexiglas and allowed to acclimate for five minutes. Once the focal female’s cylinder was removed she was allowed to swim freely for ten minutes. We recorded the time (s) females spent in both the preference zone (17.8cm) and the interaction zone (included the stimulus Plexiglas cylinder plus one body length from the stimulus females, 10.5cm).

For the preference test, we calculated the SOP scores for time spent with the stimulus females. These scores were then √arc (sin) transformed to normalize the data. We used a repeated-measures GLM to compare preference scores across the different treatments, with “treatment” and “stimulus type” being the within-subject factors. We used the age of the fish at the time of testing, the population the females originated from, and whether they had the same mother (maternal effects) as covariates. These factors were non-significant and were therefore removed from the model (Age, *F*_1,20_ = 0.415, *p* = 0.923; Population, *F*_1,18_ = 0.088, *p* = 0.916; Mother, *F*_1,20_ = 1.336, *p* = 0.291). For the shoal preference function test, we also used a repeated-measures GLM with “treatment” and “zone” as within-subject factors, and with “clone type” and “diet” as between-subject factors. We used “block” as a covariate, however, this did not have a significant effect on either the type of stimulus (*F*_4,16_ = 1.109, *p* = 0.387) or the zone (*F*_1,19_ = 0.215, *p* = 0.648) and we removed it from the model.

### Mechanism for clonal recognition: Morphometric differences

To determine the degree of visually detectable differences between clones, we assess the degree of morphology divergence among females used in the experiments. The females from the mechanism and aggression experiments (above) were sedated with MS222 after the completion of the final behavioral experiment to take several high-quality lateral photographs using a dissecting microscope (SZXZ-ILLT) and SPOT software. Several photographs were taken of both their left and right sides, and the best photograph (i.e., focus, fin position) from each was selected for further analysis. To analyze the morphology of the clonal lineages, 14 landmarks (tip of pre-maxillary, most posterior point of skull, anterior and posterior insertion points of dorsal fin, dorsal and ventral insertion points of caudal fin, anterior and posterior insertion points of anal fin, anterior insertion of pelvic fin, isthmus, dorsal and ventral insertion points of pectoral fin, dorsal most part of the opercle, and the center of eye; S6 Fig) were analyzed using geometric morphometrics (tpsDIG2) [40–43]. Once all photographs were digitized, files were converted into an nts file in order to control for the size variation among the fish (tpsUtil) [44]. A weighted matrix on the shape variables and centroid size was created using the aligned, size-corrected landmarks (tpsRelw) [45]. The shape variables and centroid size matrixes were then used in the statistical analyses [41].

Centroid size did not significantly influence the shape variables, so it was removed from the model. Also, neither focal females nor their sister clones showed significant differences for either their left (*p* = 0.152) or right (*p* = 0.497) side, hence we combined these and used “clonal lineage” as the independent variable. We used a Principle Component Analysis to reduce the number of shape variables (N = 28 for each specimen). All factors with an Eigen value of one or greater were kept [46], resulting in nine factors for both right and left side, which explained 82.315% of the variation in the right side and 79.132% in the left side. A Multivariate GLM was used to analyze the two different clonal lineages (fixed factors) with the PCA factors for the shape variables as the dependent factors.

We also investigated whether Amazon females were using body symmetry as a visual signal in clonal recognition. To test this, we used a repeated measures GLM with the “symmetry” (i.e., left and right sides) and “shape variable factors” as the within subject variables and “clone lineage” as the between subject variable. There was no significant difference between the left and right sides (F_52_ = 0.252, *P* = 0.618), the interaction of symmetry and clone lineage (F_52_ = 2.264, *P* = 0.138), the shape variable factors (F_45_ = 1.036, *P* = 0.424), the interaction of body symmetry and the shape variable factors (F_45_ = 0.348, *P* = 0.942). However, the was a significant difference between the interaction of the shape variable factors and clone lineage (F_45_ = 9.328, *P*< 0.0001), confirming the results found when the left and right side were analyzed separately.

### Kin recognition as a means to regulate aggression

Using the same females as in the above mentioned experiment investigating mechanisms, focal females were tested for their aggressive behaviors towards clonal sisters and non-sisters using two experimental designs: 1) a forced-choice (i.e., one stimulus female at a time), and 2) free-swimming (open-field) with choice (i.e., both stimulus females at the same time). Fish were given one week of rest in between the two experiments. Since these fish appear identical to the human eye, focal females had half of the dorsal fin clipped for identification. Both clonal sister and non-sister females underwent the same handling procedures as the focal female, although only one of them had their caudal fin was clipped, resulting in all three females visibly distinguishable from one another. All females were allowed to rest from handling for three days, prior to any trials.

#### Forced-Choice Experiment

Aggression was measured in a direct-contact (stimulus and focal female able the directly interact with one another) experimental tank (19L) with either a clonal sister or non-sister (S7 Fig**)**. At the start of the experiments, both focal female and stimulus female were placed in separate, clear Plexiglas cylinders. After a five-minute acclimation period females were released from the cylinders and behavioral measurements (bites, tail beats, and overall time spent being aggressive) were started at the first sign of aggression and ran for 10 minutes. We measured all three behaviors, both given to the stimulus females and received from the stimulus females. At the end of the trial both females were placed back into their individual tanks. Focal females were retested 24-hours later with the other partner, either the clonal sister or non-sister that was not tested the day before, following the same procedure. A repeated-measures GLM was employed using “Clone” and “Behavior” as the within subject factors.

#### Open Field Experiment

The open field, free-swimming aggression trials took place in a 19L experimental tank with all three females together to give the focal female a choice between the two different stimuli (S8 Fig). At the start of the experiments, both focal female and stimulus females were placed in separate clear, Plexiglas cylinders. After a 5-minute acclimation period females were released from the cylinders and behavioral measurements were started at the first sign of aggression and ran for 10-minutes. At the end of the trial all females were placed back into their individual tanks. After the completion of the experiment, females were allowed to recover and regenerate their fins. A multivariate GLM was run using “clone” as the fixed factor and the “behaviors” (bites given, tail beats given, time given, bites received, tail beats received, and time received) as the dependent variables. For both experiments, if there were no aggressive interactions among the three females after 10-minutes, the trial was terminated and the focal female was retested in 24-hours.

### Ethics statement

This research was carried out in strict accordance with the recommendations in the Guide for the Care and Use of Laboratory Animals of the National Institutes of Health. The Institutional Animal Care and Use Committee of the University of Oklahoma approved this research (#R13-006). All research was conducted at the University of Oklahoma, with exception of the natural water study, which was conducted in a hotel in Weslaco, TX. The Texas Parks and Wildlife department issued a research and collection permit # SPR-0305-045. This study did not contain any research on endangered or protected species. Fish were collected with a mesh seine and immediately transferred to an aerated cooler for transportation to the University of Oklahoma. All fish were then maintained at the Aquatic Research Facility located at the University of Oklahoma until research commenced. All efforts were made to reduce and minimize any suffering that the fish may have experienced during the course of this research. We did not experience any loss of fish, nor were any fish injured beyond the small surgical procedure of fin clipping the females for identification during the aggression study. Females that underwent the fin clipping were maintained at a higher salinity level to prevent fungal infection, and all females showed signs of fin re-growth three weeks following the surgical procedure. At the conclusion of this study all females were returned and maintained in stock populations at the OU Aquatic Research Facility.

## Results/Discussion

Using standard binary choice tests, we determined if individuals from the seven clonal lineages preferred to associate with clonal sisters to non-sisters in multiple combinations. We found that six clonal lineages exhibited a significant preference for their clonal sisters both within population lineages (CS7a –CS8b) and between population lineages (Fig 1, S7 Table), indicating that they distinguish between clonal lineages. In addition, we found no evidence to support the phenotypic matching hypothesis (i.e., the strength of discrimination does not correlate with the genetic similarity between clonal lineages, Fig 1). To determine whether this result was due to familiarity, we split sisters from one clone (CS7a) in two groups under the same conditions for over nine months (average life expectancy is 1–3 years, and sexual maturity is reached around 3 months of age) and tested the offspring of these individuals, also using standard choice tests, for their ability to recognize clonal sisters. If the recognition mechanism was based on familiarity, females should be unable to recognize unfamiliar clonal sisters. We found that females preferred clonal sisters that were unfamiliar to non-sisters (*t*_15_ = 3.362, *p* = 0.005), and familiar clonal sisters to unfamiliar clonal sisters (*t*_14_ = 2.966, *p* = 0.011; Fig 2). This result indicates that familiarity is not necessary for clonal recognition, but may strengthen the preference. Therefore, we hypothesize that a genetically based recognition mechanism for phenotype matching is adaptive for Amazon mollies. We were able to confirm our findings in a field experiment, in which wild Amazon mollies from their site of origin (Weslaco), in natural water, were allowed to choose between wild caught individuals and non-sisters from VI/17 (R = -0.264), a distant laboratory lineage. We found that wild caught females retain the ability to discriminate between clonal sisters and non-sisters in natural water (*F*_(1,19)_ = 4.926, *p* = 0.039).

**Fig 3:**
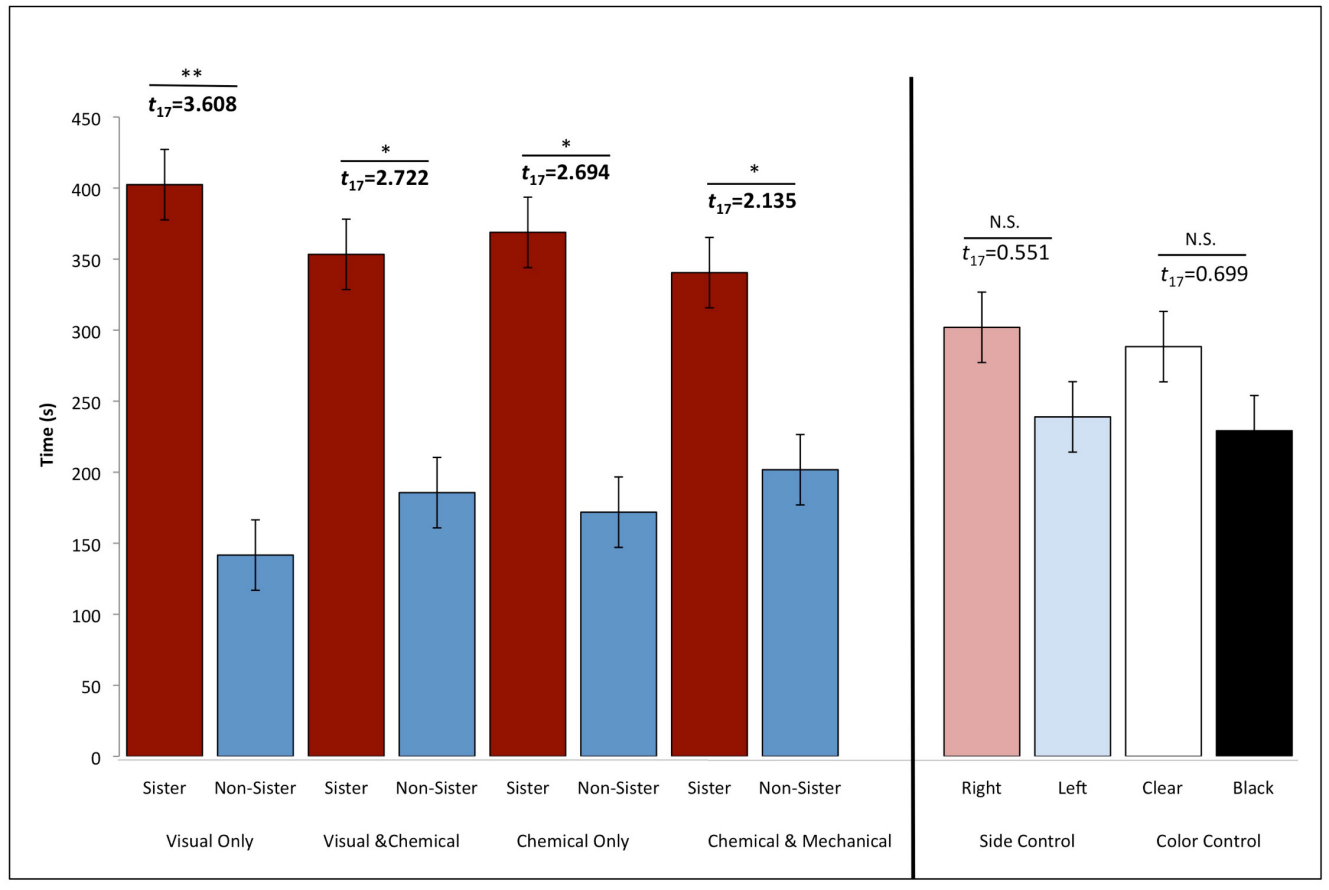
Mechanism Experiment. The average time ± SE females spent with a clonal sister (red; VI/17) and a non-sister (blue; CS7a; R = -0.057) in the four different treatments. Females showed a stronger preference to only visual signals (* = *p*<0.05; ** = *p*<0.009; NS = non-significant). The two control treatments demonstrate that there was no bias for the right (light red) or left (light blue) sides of the experimental tank and there was no bias for the clear (white) cylinder or the black (black) cylinder.

Amazon mollies show clear preferences for clonal sisters, but which sensory information is used to assess clonal identity? We concentrated on visual, chemical, and tactile information, all of which has been shown to be important in livebearing fishes. Using a repeated-measures design, we tested what cue or combination of cues (visual only, chemical only, visual and chemical cues, and chemical and mechanical cues) might be used by Amazon mollies to distinguish clonal sisters from non-sisters. All sensory modalities in isolation and in combination were sufficient for kin recognition, although there was no significant difference among sensory modalities (Mechanism: *F*_3,15_ = 0.955, *p* = 0.439; Fig 3). Within each modality, post-hoc analyses indicate that females showed the strongest preference for clonal sisters when only visual cues were present; nonetheless, they still showed a significant preference when only chemical cues, a combination of chemical/mechanical, or visual/chemical cues were presented. Female activity, however, was higher when chemical cues were present, and they entered the preference zones that included the clonal sisters more often (*F*_1,17_ = 8.285, *p* = 0.010). Although it is known that Amazons prefer conspecific females when compared to their heterospecific host even when chemical only cues are present [30], here we show that their discriminatory abilities are even more precise than previously thought. In addition, we found no difference in the strength of kin recognition in the presence of unimodal and bimodal cues (*F*_1,70_ = 1.256, *p* = 0.266), suggesting that discrimination is not improved using more than one sensory channel. This lends support to the conclusion that signals are often redundant, conveying comparable cues [47]. Most importantly, we were able to find the same effect in natural water. As in the laboratory, wild caught females preferred clonal sisters when chemical information was available (*t*_(19)_ = 3.805, *p* = 0.001), while the preference using visual information was not detectable (*t*_(19)_ = 0.310, *p* = 0.760; S9 Fig). This was likely due to naturally high turbidity of the water [48] as Amazon mollies are found in both turbid and clear environments and it is possible that they may rely more on either visual or chemical cues depending on the environment they live in.

**Fig 4:**
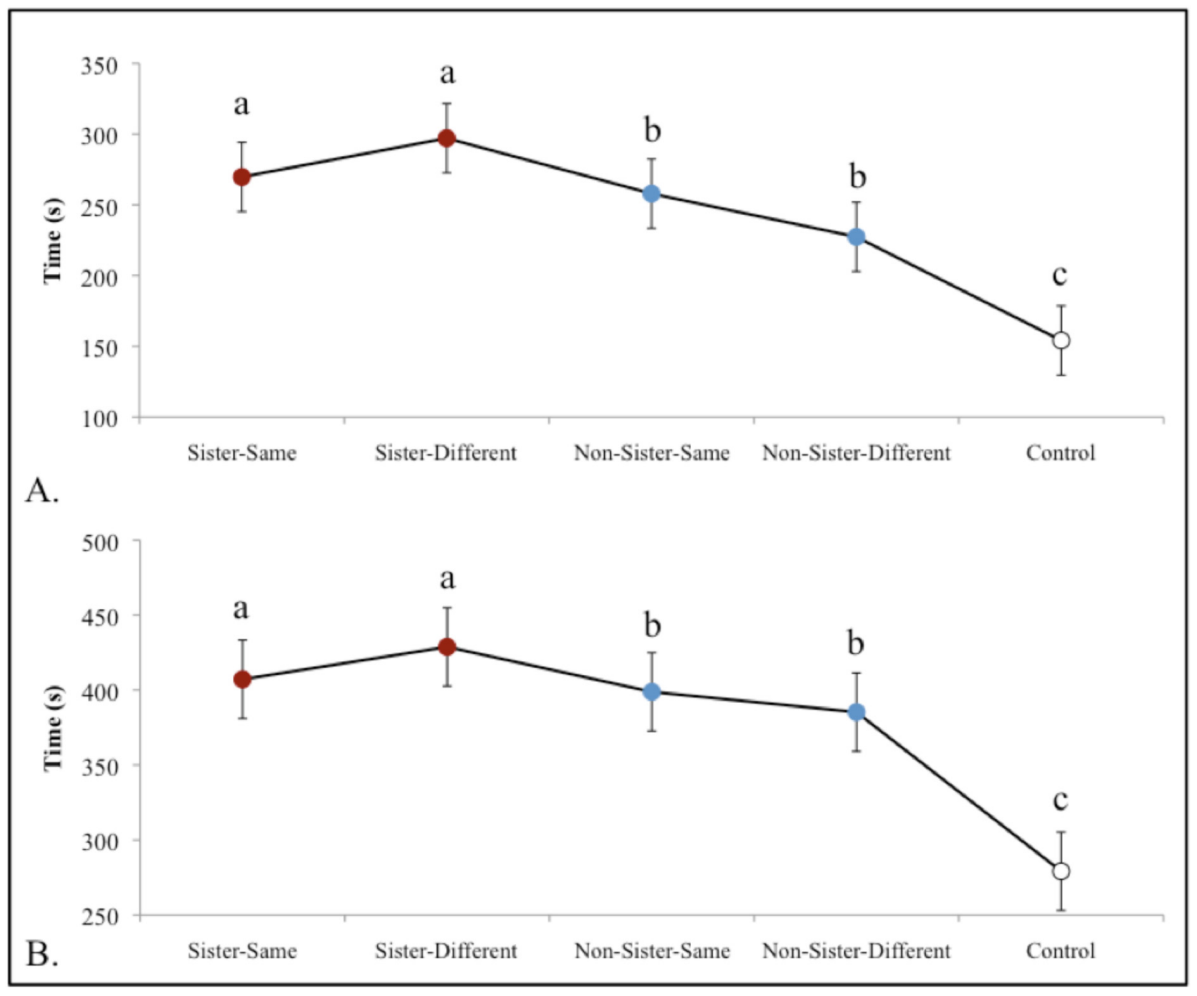
Shoaling Preference Experiment. Average time (s) ± SE females spent near the stimulus females in (**A.**) the interacting zone and (**B.**) the preference zone. Females spent significantly more time with the clonal sisters (red) on a different diet in both zones (preference zone: *t*_(23)_ = 2.792, *p* = 0.010; interaction zone: *t*_(23)_ = 2.909, *p* = 0.008) when compared to non-sisters (blue) on a different diet. They also tended to spend more time with clonal sisters on a different diet to non-sisters on the same diet (preference zone: *t*_(23)_ = 2.027, *p* = 0.054), and with clonal sisters in general, regardless of diet, that with non-sisters (interaction zone: *t*_(47)_ = 2.132, *p* = 0.038).

Nonetheless, there are various visual (i.e., body shape, pigment cell quantity and expression, etc.) and chemical cues (dietary, MHC genes, maternally inherited micro-biomes, etc.) in which clones may differ. Using geomorphometric analysis, we investigated body shape as a potential visual cue, and found females from clone CS7a to be significantly different from the females from clone VI/17 in body shape (Right: F_44_ = 9.592, *p*< 0.0001; Left: F_44_ = 6.235, *p*< 0.0001; S10 and S11 Figs). Overall, Amazon females from clone CS7a had deeper bodies, a more terminal mouth, a larger head, and a slightly longer and deeper caudal-peduncle. Body symmetry, however, did not differ between the clonal lineages (F_52_ = 2.264, *P* = 0.138). A previous study in *Gambusia hubbsi*, suggest that individuals can distinguish between very small differences in the body shape of conspecifics, and that this significantly influences an individual’s preference [42]. This suggests that we cannot rule out body shape as a potential mechanism for visual clonal recognition. For a potential chemical signal, we evaluated how diets may influence individual preference via chemical only cues using a common garden experimental design. We found that inexperienced females retain a preference for clonal sisters on a different diet over non-sisters on the same diet (*t*_33_ = 3.643, *p* = 0.001) and prefer to spend more time interacting with clonal sister shoals, regardless of the diet they were on, as compared to non-sister shoals (Fig 4). This suggests that diet alone is not sufficient to alter kin recognition in these fish.

**Fig 5:**
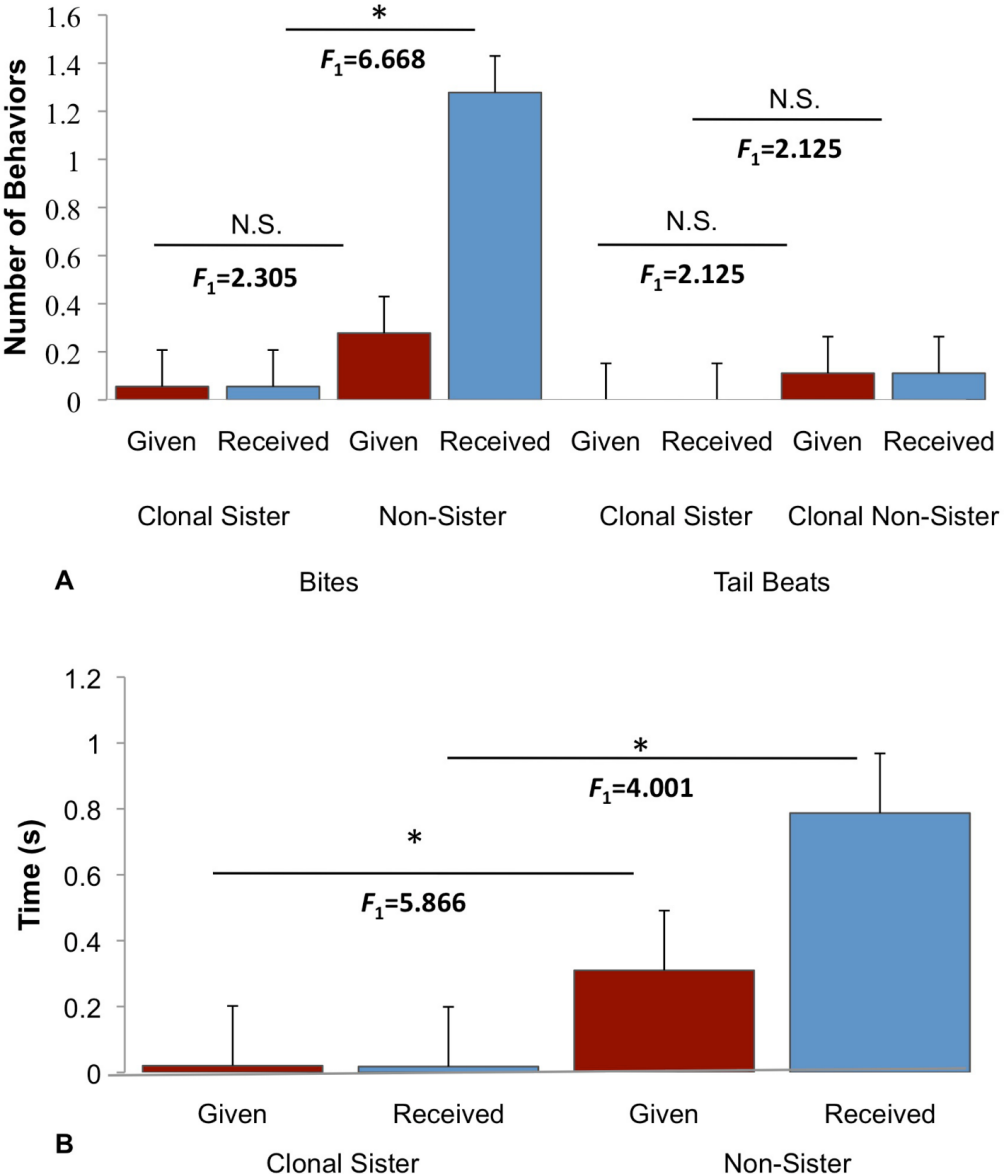
Aggression Experiment. This experiment tested the aggression levels of females when given a choice between a clonal sister (VI/17 Río Purificación, Nuevo Padilla, MX) and non-sister (Comal Spring, TX (7a), (Relatedness coefficient = -0.057; average±SE). Females received (blue) significantly more bites (**A.** given: *F*_(1)_ = 2.305, *p* = 0.138; received: *F*_(1)_ = 6.668, *p* = 0.014) and spent significantly more time performing (given = red) aggressive behaviors (**B.** given: *F*_(1)_ = 5.866, *p* = 0.021; received: *F*_(1)_ = 4.001, *p* = 0.054) towards non-sisters when compared to clonal sisters. There was no significant difference in performing tail beats (**A.** given: *F*_(1)_ = 2.125, *p* = 0.154; received: *F*_(1)_ = 2.125, *p* = 0.154).

The presence of clonal recognition in an asexual vertebrate is interesting in itself, but a key question is: what adaptive benefit might Amazon mollies derive from kin recognition? Due to intraspecific competition and the extensive niche overlap between Amazons and their sexual hosts, we hypothesized that females may show more aggression towards non-sisters (and heterospecific sexual females) than clonal sisters to acquire access to limited resources, like food and potential mates [24,49–50]. Indeed, aggression in Amazon mollies has been shown to decrease their overall fitness via lower body fat condition and increasing energy expenditure [31]. We designed an open field experiment measuring the aggressive behaviors of females that were allowed to interact with both a clonal sister and non-sister. Females behaved more aggressively towards non-sisters (*F*_6,29_ = 2.490, *p* = 0.046; Fig 5), as would be predicted if clonal recognition is used in regulation of aggression. We also conducted a forced-choice experiment where females were allowed to interact with either a clonal sister or non-sister, which showed similar results (*F*_1,17_ = 8.981, *p* = 0.002; S2 Fig). Together, this suggests that it is adaptive for Amazon mollies to regulate their aggressive behaviors towards clonal sisters and non-sisters due to the high cost incurred on their fitness via reduced body conditioning and potential energy available to invest into future offspring [31].

In sexual species, kin recognition can evolve between closely related and distantly related individuals that are more genetically distinct (i.e., individuals share either 50% or 25% of genes that are identical by descent). It is likely to evolve when siblings overlap in time and space, and are able to recognize each other independently of context and familiarity [3]. The same parameters would hold true for asexual species; however, there is a much smaller genetic difference between individuals, which may suggest relaxed selection on other preferences. Alternatively, with female clones being genetically identical to one another, females may be using self-referential phenotype matching (using one’s own cues as a reference in discriminating between kin and non-kin), which would perceptually be an easier task as compared to a sexual species. Although we did find that familiarity was present but not necessary, this may suggest that there are multiple cues in which lead to more precise discrimination between clonal sisters and non-sisters. Other factors such as maternal or epigenetic effects may also contribute to the phenotypic diversification between the different clonal lineages. However, given the controlled environment in which all the females were raised and maintained, this is an unlikely explanation for our results. Although it is known that diet and gut microbiota may influence the ability to recognize kin [51–52], we raised juveniles on different diets, manipulating their chemical signals and the gut microbiota, and found that juveniles were still able to recognize and prefer clonal sisters regardless of diet type and maternal effects. Nonetheless, epigenetics are of great interest when investigating clonal species.

With their ability for clonal recognition, Amazon mollies are one of the most extreme examples corroborating the predictions of kin selection theory, where aggression is regulated in a way that extremely close (i.e., genetically identical) kin are favored over very close kin. Given the substantial genetic similarity found throughout the whole species [20–21], up to 15 genetically distinct clonal lineages have been found within a single population with each lineage varying in frequency [AM Makowicz, unpublished data]. Nonetheless, this indicates that it is likely beneficial for clones to be able to recognize each other and regulate competition in a way that favors extremely close kin; even minute genetic differences provide enough substrate for kin recognition. We believe that the discrimination ability found in Amazons could be a powerful example of natural selection in action.

## Supporting Information

S1 Fig Map of Population Localities. Six populations (red dots) of *P*. *formosa* were used in the female preference study: San Marcos (County 101), Comal Spring, Weslaco, San Ignacio, VI/17, and III/9. These populations are part of four different river drainage basins across Texas and Mexico (Guadalupe (orange; San Marcos (County 101) and Comal Spring), Río Grande (green; Weslaco), Río San Fernando (purple; San Ignacio), and Río Pánuco (blue; VI/17 and III/9)), and cover the geographical distribution of *P*. *formosa* (both introduced populations in central Texas, and native range in South Texas and East Mexico).
(PDF)

S2 FigAggression Experiment #1: Forced Choice.Female aggression was tested in a forced choice design to measure the baseline aggression levels toward clonal sisters and non-sisters for each female ($\bar{\chi }\pm \text{SE}$). Females gave (red) and received (blue) significantly more bites (**A.** given: *t*_(17)_ = -2.715, *p* = 0.015; received: *t*_(17)_ = -3.308, *p* = 0.004), and spent more time being aggressive (**B.** given: *t*_(17)_ = -2.078, *p* = 0.053; received: *t*_(17)_ = -2.330, *p* = 0.032) towards non-sisters when compared to clonal sisters. There was no significant difference in performing tail beats (given: *t*_(17)_ = -1.000, *p* = 0.331; received: *t*_(17)_ = -1.000, *p* = 0.331).(PDF)

S3 Fig Female Preference Experimental Set-up. Standard choice test allowing visual, chemical and mechanical cues, where the focal female swims freely between both stimuli. The dashed circles represent the clear, perforated cylinders and the dashed lines represent the preference zones. The amount of time (s) she spends in each zone reflects a preference for the stimulus in that zone. This set-up was used in the initial female preference experiment and familiarity experiment. It was then adjusted for the mechanism experiment, where the dashed circles selectively excluded selected mechanism pre-treatment requirements.
(PDF)

S4 Fig Plexiglas Cylinder Construction and Functions. We tested the construction of each Plexiglas cylinder to validate its proper construction and function. Each picture illustrates the diffusion of water from inside each of the 4 Plexiglas cylinders to the preference zone (**A**. visual only cues; **B**. visual and chemical cues; **C**. chemical only cues (each perforated hole had a small area, about 7 mm^2^, to reduce hair cell stimulation); and **D**. chemical and mechanical cues (each perforated hole had a large area, about 20 mm^2^, to allow hair cell stimulation)). A female was placed inside each cylinder to provide normal water disturbance, food coloring was added to the cylinders, then set-up was recorded for the full 10-minute acclimation period. Diffusion usually occurred within 2–4 minutes after food coloring was added, demonstrating that female chemical cues would be present in the preference zones after the 10-minute acclimation period.
(PDF)

S5 Fig Shoaling Preference Experimental Tank. This represents a diagram of the experimental preference function test used to evaluate the overall time spent with the four different shoal types. The thick solid black line represents the clear, perforated Plexiglas cylinder that the stimulus shoal was kept in. The first dashed line (A.) represents the interaction zone (10.5 cm) and the second dashed line (B.) represents the preference zone (17.8 cm).
(PDF)

S6 Fig Morphological landmarks. The 14 landmarks used in the geometric morphometrics depicted on a photo of a *Poecilia formosa* female. 1) tip of pre-maxillary, 2) most posterior point of skull, 3) anterior and 4) posterior insertion points of dorsal fin, 5) dorsal and 6) ventral insertion points of caudal fin, 7) posterior and 8) anterior insertion points of anal fin, 9) anterior insertion of pelvic fin, 10) isthmus, 11) ventral and 12) dorsal insertion points of pectoral fin, 13) dorsal most part of the opercle, and 14) the center of eye.
(PDF)

S7 Fig Female Aggression Experimental set-up #1: Forced Choice. This figure is a representation of the experimental tank for the female aggression experiment to test without a choice. The experiment was used to measure the baseline aggression levels toward clonal sister and non-sister for each female. Females were placed into clear, perforated Plexiglas cylinders (dashed circle) in the centre of the tank along with a stimulus female, either a clonal sister or a non-sister. Females were released after a 10-minute acclimation period, allowed to swim freely and interact with each other. Aggressive behaviors and overall time spent (s) being aggressive was recorded for the duration of 10 minutes. Females were the tested again with the other stimulus female after 24 hours.
(PDF)

S8 Fig Female Aggression Experimental set-up #2: Free-swimming with Choice. This figure is a representation of the experimental tank used to test the aggression levels when females are given a choice between either clonal sisters or non-sisters. Females were placed into clear, perforated Plexiglas cylinders (dashed circle) in the centre of the tank along with stimulus females, a clonal sister and non-sister. Females were released after a 10-minute acclimation period, allowed to swim freely and interact with each other. Aggressive behaviors and overall time spent (s) being aggressive was recorded for the duration of 10 minutes.
(PDF)

S9 Fig Field study of clonal recognition. We tested clonal recognition for both visual signals only and chemical signals only in a naturally turbid stream located in Weslaco, TX. We found that females loose the ability to discriminate between clonal sisters (red; VI/17 Río Purificación, Nuevo Padilla, MX) and non-sisters (blue; Comal Spring, TX (7a), (Relatedness coefficient = -0.057) when only visual signals are available, most likely due to the naturally high turbidity. On the other hand, females were able to recognize clonal sisters compared to nonsisters when only chemical signals were present.
(PDF)

S10 Fig Geometric morphometrics at normal resolution (1X). Body shape of both the Amazon mollies from the Mexico population (top, focal and sister clones, red), and the Texas population (middle, non-sister clones, blue). The bottom demonstrates the differences in morphology between the two populations by overlaying of both body shapes to show the minute differences in morphology. Overall, morphology was significantly different between Amazons from the Texas and Mexico population (Right: *F*_(44)_ = 9.592, *p*< 0.0001; Left: *F*_(44)_ = 6.235, *p*< 0.0001). Females from Texas had deeper bodies, a more terminal mouth, a larger head, and a slightly longer and deeper caudal-peduncle.
(PDF)

S11 Fig Geometric morphometrics at three-times the resolution (3X). Body shape of both the Amazon mollies from the Mexico population (top, focal and sister clones, red), and the Texas population (middle, non-sister clones, blue). The bottom demonstrates the exaggerated differences in morphology between the two populations by overlaying of both body shapes to show the minute differences in morphology.
(PDF)

S1 Table Population origins. This table shows the test population origins across the range of *P*. *formosa*, indicating the location, drainage basin and the coordinates of the original population collection site). There were 2 populations from the northern range (San Marcos (C101) and Comal Spring), 2 populations from the midpoint (Weslaco and San Ignacio), and 2 populations from the southern range (Río Purificacíon, Barretal (III/9) and Río Purificacíon, Nuevo Padilla (VI/17). Note: the populations sampled covered the span of the geographical distribution of *P*. *formosa*.
(PDF)

S2 Table Characteristics of the 12 microsatellites. Here, summary statistics of the 12 microsatellites that were used in differentiating the 7 different clonal lineages of *P*. *formosa* are shown, i.e., population, sample size, the number of alleles, the observed heterozygosity of the current generation (H_0_), the expected heterozygosity (H_E_), the probability of Hardy-Weinberg-Equilibrium (HWE), i.e., H_0_ = H_E_ (P), and the F_ST_ value of all populations at that particular locus. Note that loci are generally expected not to be in HWE in Amazon mollies, due to the lack of sexual recombination.
(PDF)

S3 Table Genetic divergence. Genetic divergence among the 7 clonal lineages of *P*. *formosa*. Above the diagonal are the F_ST_ values and below the diagonal are the *P*-values from the Markov Chain Monte Carlo exact test. Statistical significance after sequential Bonferroni correction for multiple pairwise comparisons is indicated at an experiment-wise error rate α (*α = 0.05; **α = 0.01; *** α = 0.001).
(PDF)

S4 Table Loci difference and geographic range. This table shows the geographical distance (km) between the 7 different clonal lineages above the diagonal. Below the diagonal are the number of loci that are different in their allelic pattern between the different clonal lineages. Interestingly, the San Ignacio clonal lineages is located 552.8 km south of Comal Spring, yet the Comal Spring 7a lineage is identical to the San Ignacio lineage (for the 12 microsatellites that we tested them for) and not to a sympatric clonal lineage Comal Spring 8b.
(PDF)

S5 Table Genetic Identity. The genetic identity within each clonal lineage is higher (i.e., more closely related among each other; 1.000 = 100% genetically identical within clonal lineage) than between clonal lineages, with exception of C101. The lack of higher genetic identity with the C101 clonal lineage may be reflected in the lack of behavioral evidence for kin recognition in this clonal lineage.
(PDF)

S6 Table Relatedness Coefficient. The coefficient of relatedness within each clonal lineage is higher than between clonal lineages, with exception of C101. R = 1: identical twins/clones; R = 0.5: clonal populations as related to each other as full siblings would be in an outcrossing, sexual species; and R<0: less identity than at random (i.e., individuals are as dissimilar to each other as unrelated individuals would be in outcrossing, sexual species with the lower numbers indicating the more unlikely related the lineages are). Note: the underlying logic of R is assuming sexual reproduction of diploid organisms, and therefore, these values are only considered an approximation in clonal organisms.
(PDF)

S7 Table Kin Preference Scores From Multiple Populations and Pairings. The strength of preference score (SOP) for several different clonal pairs including: the focal population’s genetic identity; the relatedness coefficient between the focal population and the non-sister stimulus population; the raw preference (s), average SOP and standard deviation for a preference towards the clonal sisters; the raw preference (s), average SOP and standard deviation for a preference towards the non-sister clones; the geographical distance between the two population lineages; and the sample size, t-score, and p-value from the t-tests. Pairs were familiar clonal sister and unfamiliar non-sister, unfamiliar clonal sister and unfamiliar non-sister, or familiar clonal sister and unfamiliar clonal sister.
(PDF)
