# Supplementary figures and images for: Kin Recognition in a Clonal Fish, Poecilia formosa
Source: PLoS One. 2016 Aug 2;11(8):e0158442. doi: 10.1371/journal.pone.0158442 (PMC4970819; doi:10.1371/journal.pone.0158442)

**S1 Figure**

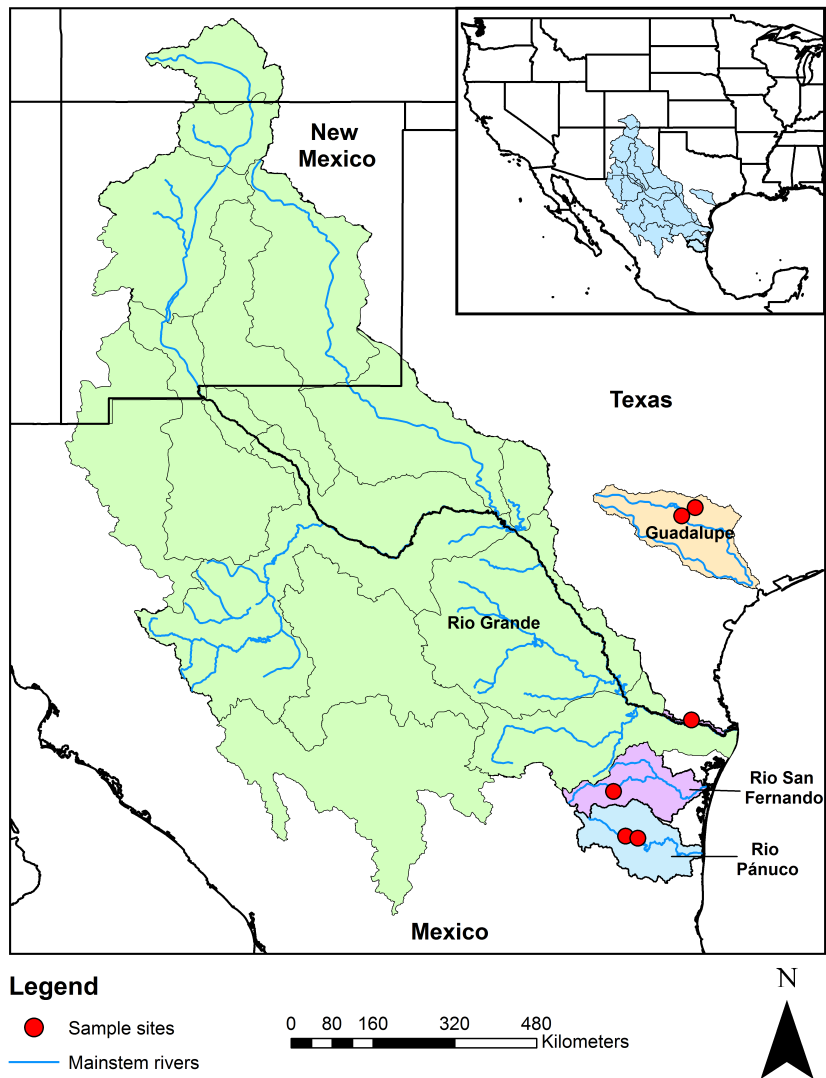

Supplement: S1 Fig — Six populations (red dots) of P. formosa were used in the female preference study: San Marcos (County 101), Comal Spring, Weslaco, San Ignacio, VI/17, and III/9. These populations are part of four different river drainage basins across Texas and Mexico (Guadalupe (orange; San Marcos (County 101) and Comal Spring), Río Grande (green; Weslaco), Río San Fernando (purple; San Ignacio), and Río Pánuco (blue; VI/17 and III/9)), and cover the geographical distribution of P. formosa (both introduced populations in central Texas, and native range in South Texas and East Mexico). (PDF) [file pone.0158442.s001.pdf]

**S2 Figure**

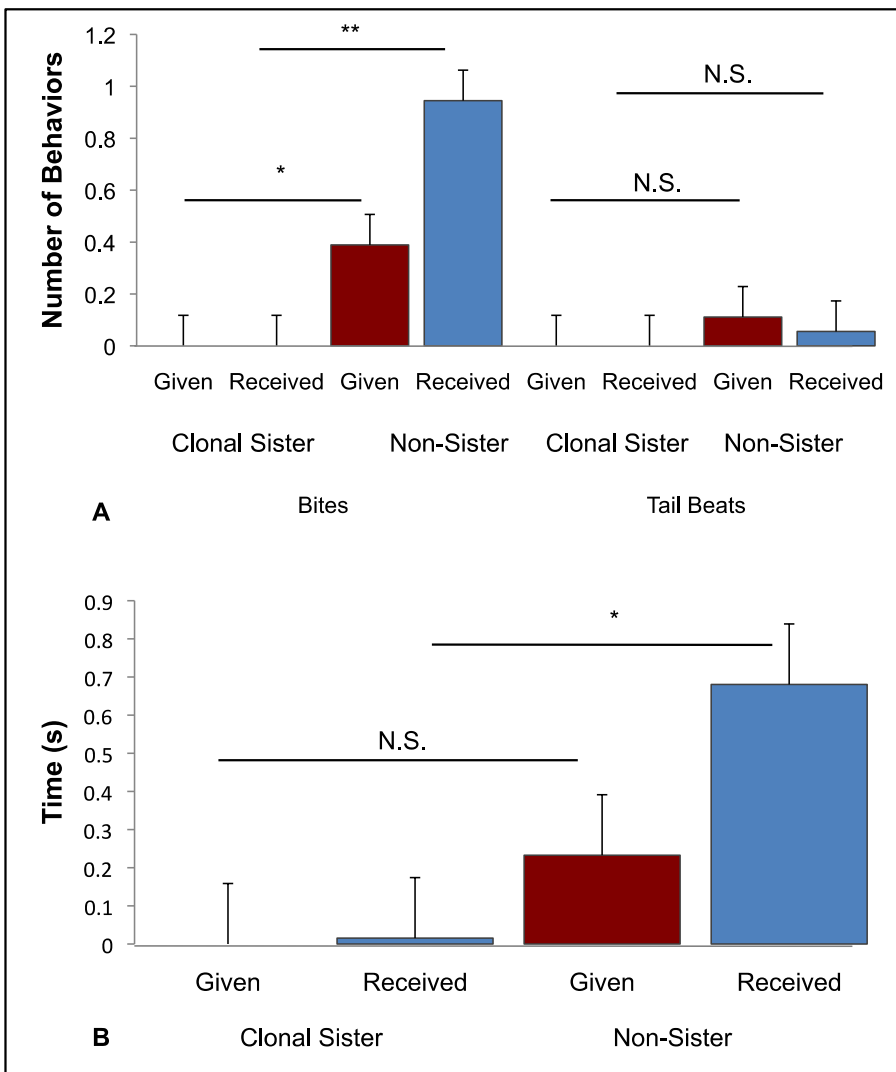

Supplement: S2 Fig — Female aggression was tested in a forced choice design to measure the baseline aggression levels toward clonal sisters and non-sisters for each female (χ¯±SE). Females gave (red) and received (blue) significantly more bites (A. given: t(17) = -2.715, p = 0.015; received: t(17) = -3.308, p = 0.004), and spent more time being aggressive (B. given: t(17) = -2.078, p = 0.053; received: t(17) = -2.330, p = 0.032) towards non-sisters when compared to clonal sisters. There was no significant difference in performing tail beats (given: t(17) = -1.000, p = 0.331; received: t(17) = -1.000, p = 0.331). (PDF) [file pone.0158442.s002.pdf]

**S3 Figure**

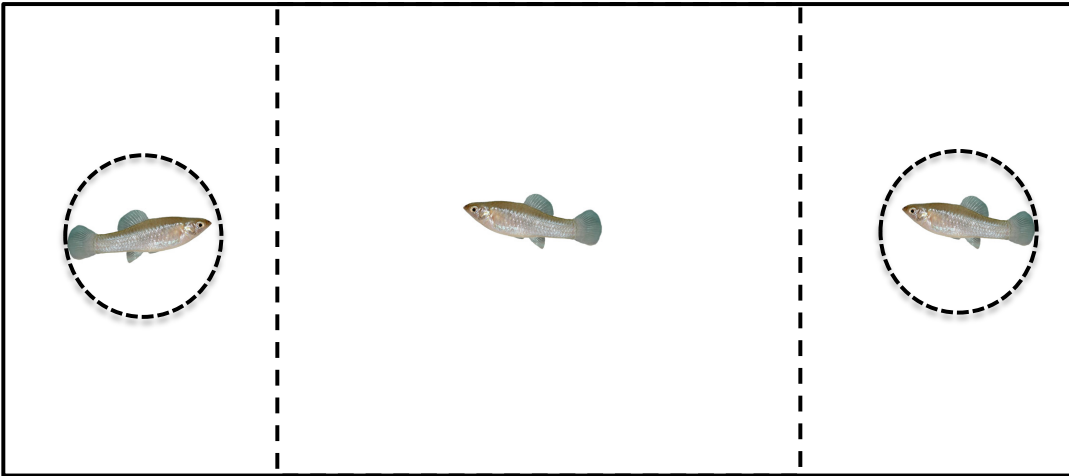

Supplement: S3 Fig — Standard choice test allowing visual, chemical and mechanical cues, where the focal female swims freely between both stimuli. The dashed circles represent the clear, perforated cylinders and the dashed lines represent the preference zones. The amount of time (s) she spends in each zone reflects a preference for the stimulus in that zone. This set-up was used in the initial female preference experiment and familiarity experiment. It was then adjusted for the mechanism experiment, where the dashed circles selectively excluded selected mechanism pre-treatment requirements. (PDF) [file pone.0158442.s003.pdf]

**S4 Figure**

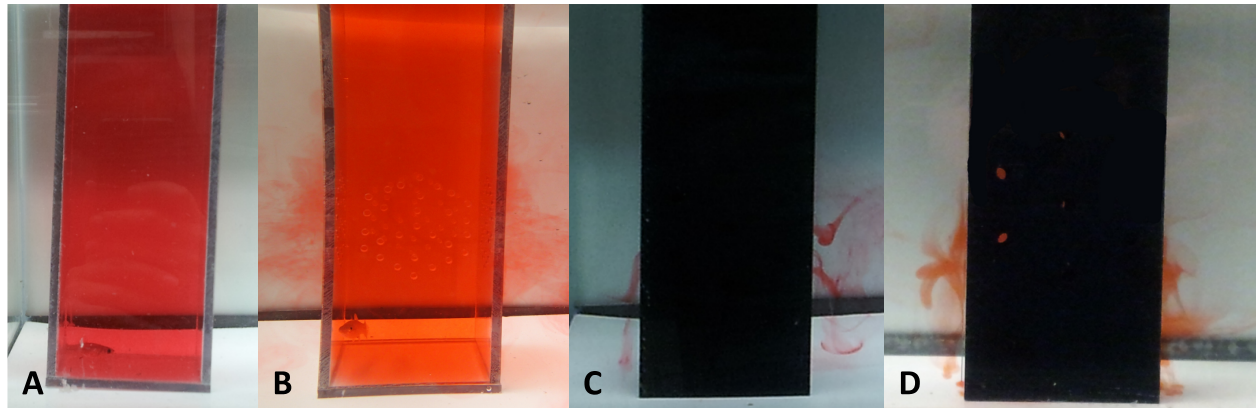

Supplement: S4 Fig — We tested the construction of each Plexiglas cylinder to validate its proper construction and function. Each picture illustrates the diffusion of water from inside each of the 4 Plexiglas cylinders to the preference zone (A. visual only cues; B. visual and chemical cues; C. chemical only cues (each perforated hole had a small area, about 7 mm2, to reduce hair cell stimulation); and D. chemical and mechanical cues (each perforated hole had a large area, about 20 mm2, to allow hair cell stimulation)). A female was placed inside each cylinder to provide normal water disturbance, food coloring was added to the cylinders, then set-up was recorded for the full 10-minute acclimation period. Diffusion usually occurred within 2–4 minutes after food coloring was added, demonstrating that female chemical cues would be present in the preference zones after the 10-minute acclimation period. (PDF) [file pone.0158442.s004.pdf]

S5 Figure

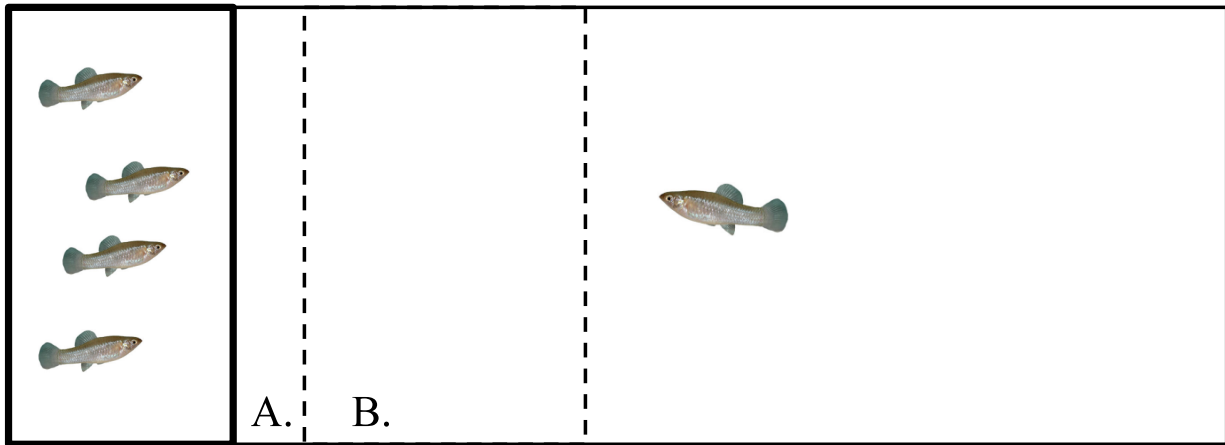

Supplement: S5 Fig — This represents a diagram of the experimental preference function test used to evaluate the overall time spent with the four different shoal types. The thick solid black line represents the clear, perforated Plexiglas cylinder that the stimulus shoal was kept in. The first dashed line (A.) represents the interaction zone (10.5 cm) and the second dashed line (B.) represents the preference zone (17.8 cm). (PDF) [file pone.0158442.s005.pdf]

S6 Figure

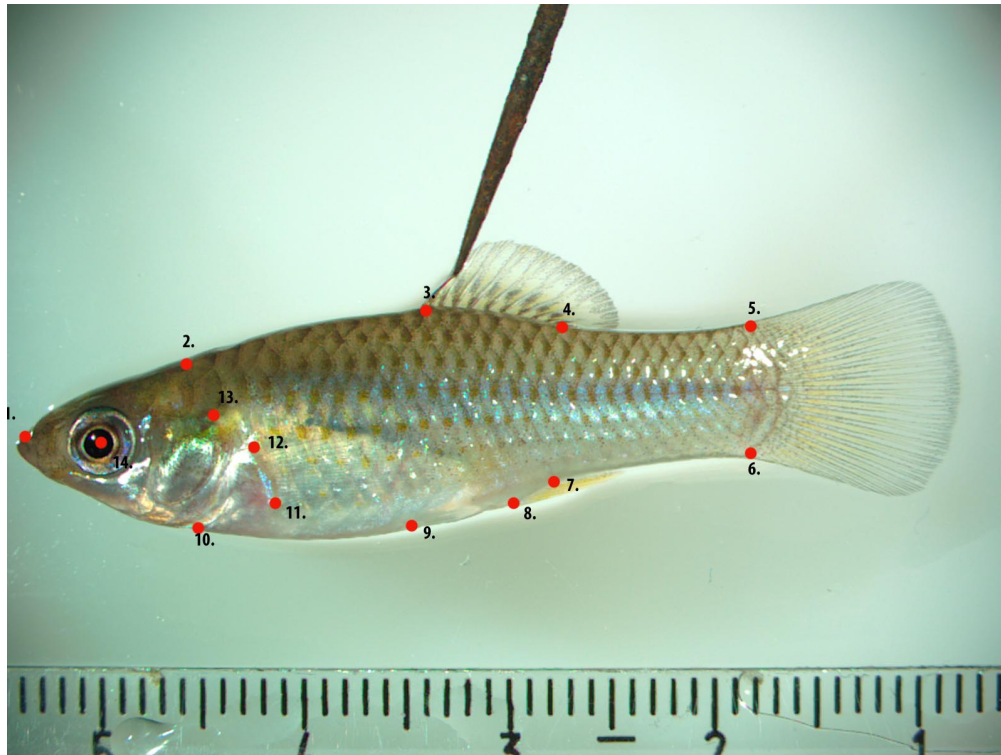

Supplement: S6 Fig — The 14 landmarks used in the geometric morphometrics depicted on a photo of a Poecilia formosa female. 1) tip of pre-maxillary, 2) most posterior point of skull, 3) anterior and 4) posterior insertion points of dorsal fin, 5) dorsal and 6) ventral insertion points of caudal fin, 7) posterior and 8) anterior insertion points of anal fin, 9) anterior insertion of pelvic fin, 10) isthmus, 11) ventral and 12) dorsal insertion points of pectoral fin, 13) dorsal most part of the opercle, and 14) the center of eye. (PDF) [file pone.0158442.s006.pdf]

**S6 Figure**

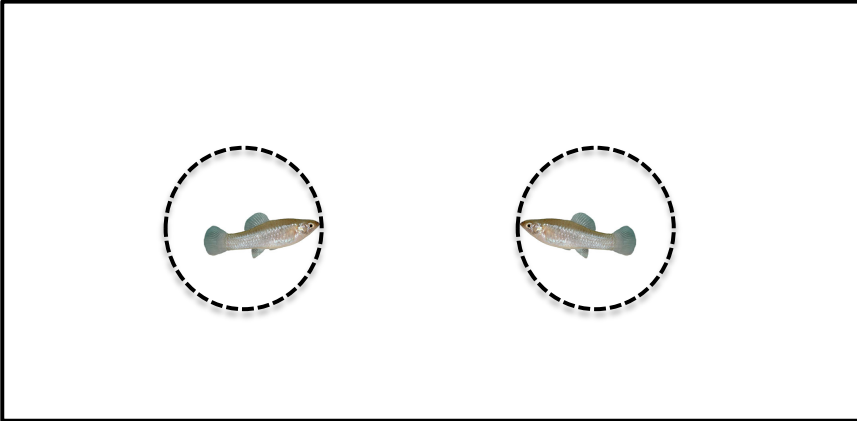

Supplement: S7 Fig — This figure is a representation of the experimental tank for the female aggression experiment to test without a choice. The experiment was used to measure the baseline aggression levels toward clonal sister and non-sister for each female. Females were placed into clear, perforated Plexiglas cylinders (dashed circle) in the centre of the tank along with a stimulus female, either a clonal sister or a non-sister. Females were released after a 10-minute acclimation period, allowed to swim freely and interact with each other. Aggressive behaviors and overall time spent (s) being aggressive was recorded for the duration of 10 minutes. Females were the tested again with the other stimulus female after 24 hours. (PDF) [file pone.0158442.s007.pdf]

**S7 Figure**

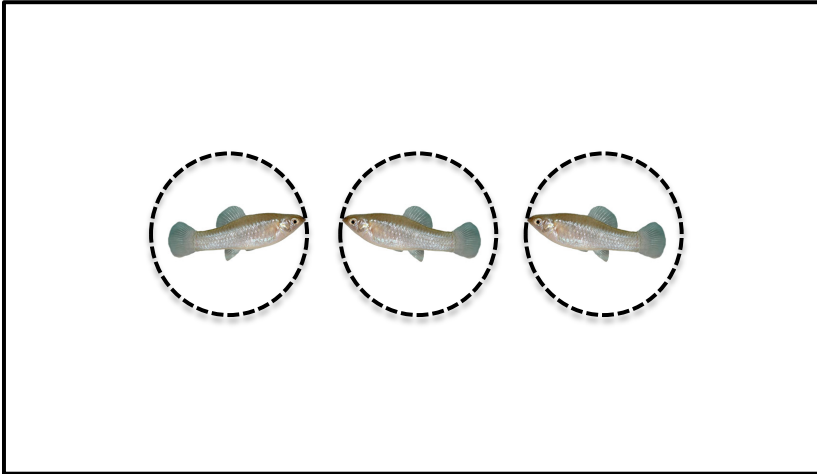

Supplement: S8 Fig — This figure is a representation of the experimental tank used to test the aggression levels when females are given a choice between either clonal sisters or non-sisters. Females were placed into clear, perforated Plexiglas cylinders (dashed circle) in the centre of the tank along with stimulus females, a clonal sister and non-sister. Females were released after a 10-minute acclimation period, allowed to swim freely and interact with each other. Aggressive behaviors and overall time spent (s) being aggressive was recorded for the duration of 10 minutes. (PDF) [file pone.0158442.s008.pdf]

**S9 Figure**

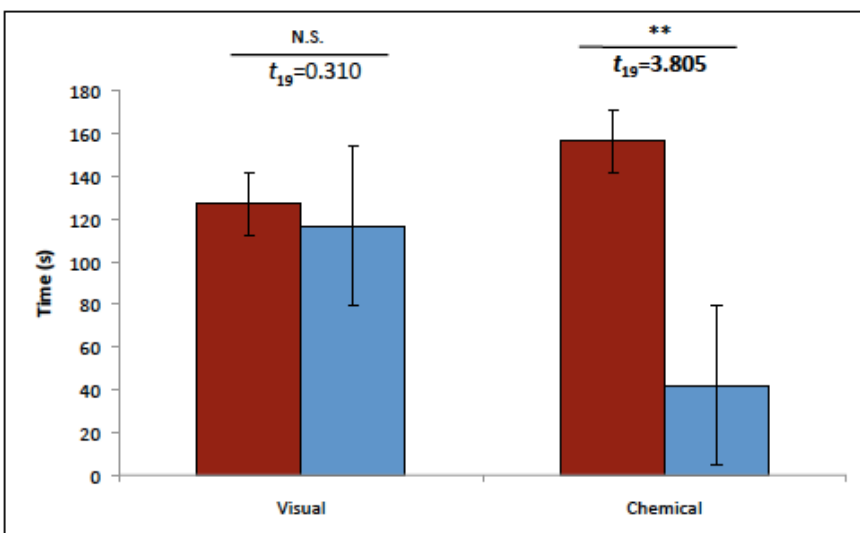

Supplement: S9 Fig — We tested clonal recognition for both visual signals only and chemical signals only in a naturally turbid stream located in Weslaco, TX. We found that females loose the ability to discriminate between clonal sisters (red; VI/17 Río Purificación, Nuevo Padilla, MX) and non-sisters (blue; Comal Spring, TX (7a), (Relatedness coefficient = -0.057) when only visual signals are available, most likely due to the naturally high turbidity. On the other hand, females were able to recognize clonal sisters compared to nonsisters when only chemical signals were present. (PDF) [file pone.0158442.s009.pdf]

S10 Figure

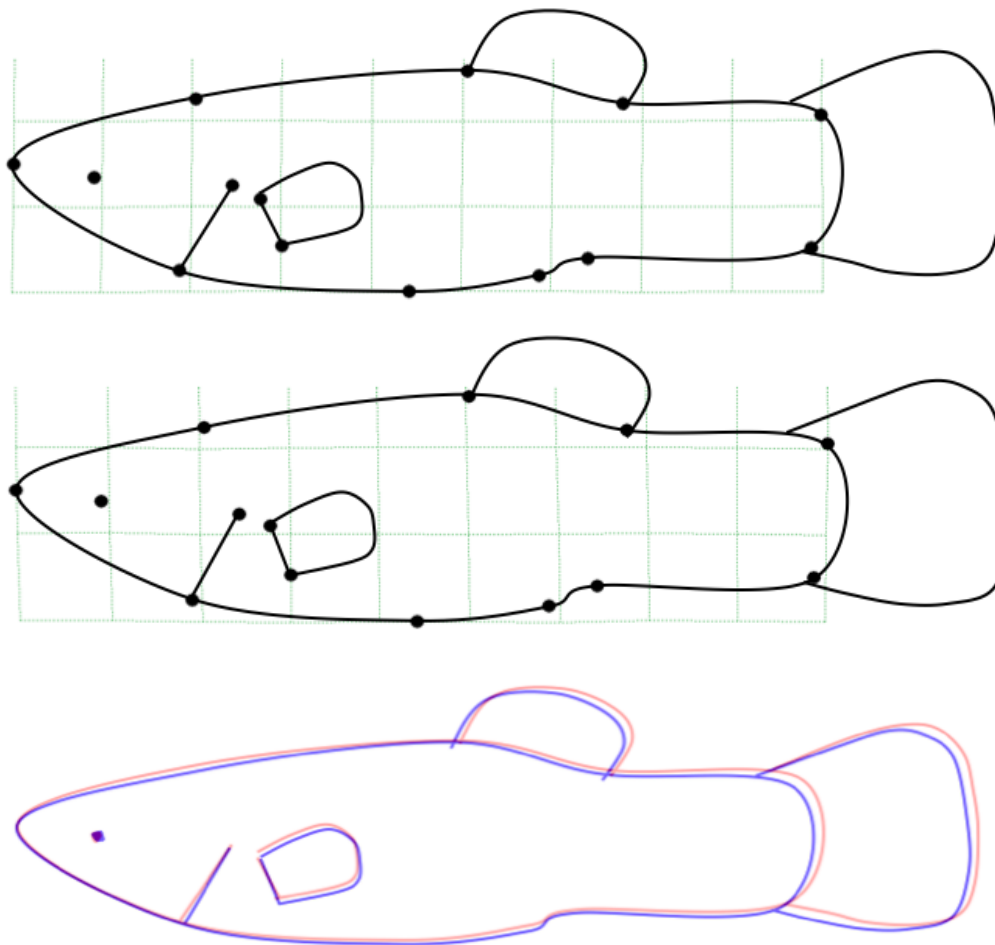

Supplement: S10 Fig — Body shape of both the Amazon mollies from the Mexico population (top, focal and sister clones, red), and the Texas population (middle, non-sister clones, blue). The bottom demonstrates the differences in morphology between the two populations by overlaying of both body shapes to show the minute differences in morphology. Overall, morphology was significantly different between Amazons from the Texas and Mexico population (Right: F(44) = 9.592, p< 0.0001; Left: F(44) = 6.235, p< 0.0001). Females from Texas had deeper bodies, a more terminal mouth, a larger head, and a slightly longer and deeper caudal-peduncle. (PDF) [file pone.0158442.s010.pdf]

S11 Figure

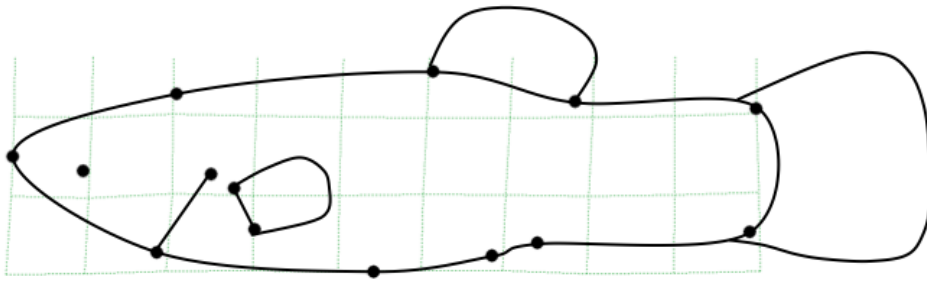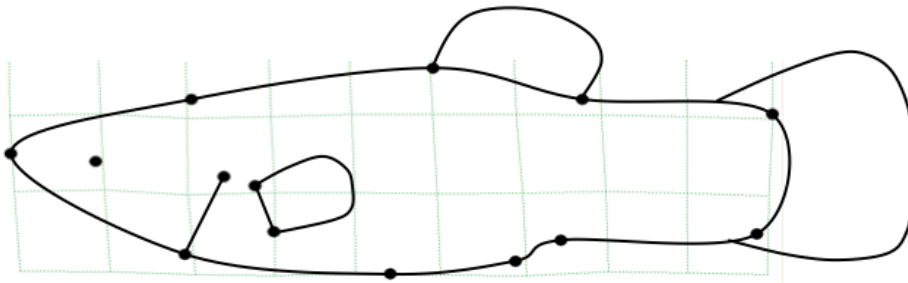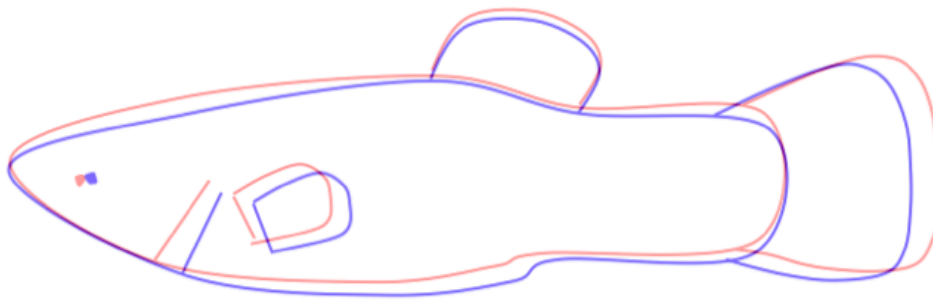

Supplement: S11 Fig — Body shape of both the Amazon mollies from the Mexico population (top, focal and sister clones, red), and the Texas population (middle, non-sister clones, blue). The bottom demonstrates the exaggerated differences in morphology between the two populations by overlaying of both body shapes to show the minute differences in morphology. (PDF) [file pone.0158442.s011.pdf]
