# Supplementary material for: Kin Recognition in a Clonal Fish, Poecilia formosa
Source: PLoS One. 2016 Aug 2;11(8):e0158442. doi: 10.1371/journal.pone.0158442 (PMC4970819; doi:10.1371/journal.pone.0158442)
Supplement: S1 Table — This table shows the test population origins across the range of P. formosa, indicating the location, drainage basin and the coordinates of the original population collection site). There were 2 populations from the northern range (San Marcos (C101) and Comal Spring), 2 populations from the midpoint (Weslaco and San Ignacio), and 2 populations from the southern range (Río Purificacíon, Barretal (III/9) and Río Purificacíon, Nuevo Padilla (VI/17). Note: the populations sampled covered the span of the geographical distribution of P. formosa. (PDF) [file pone.0158442.s012.pdf]

S1 Table.

Tested Population	Location	Drainage Basin	Coordinates	
			North	West
San Marcos (C101)	Texas, USA	Guadalupe River	29°51'25.83	97°53'47.96
Comal Spring	Texas, USA	Guadalupe River	29°42'46.82	98°8'8.25
Weslaco	Texas, USA	Río Grande	26°7'14.52	97°57'41.44
Río Purificacíon, Barretal (III/9)	Mexico	Río Pánuco	24°4'42.85	99°7'21.76
Río Purificacíon, Nuevo Padilla (VI/17)	Mexico	Río Pánuco	24°02'35.59	98°54'15.98
San Ignacio	Mexico	Río San Fernando	24°51'53.2	99°20'02.7
