# Supplementary material for: Kin Recognition in a Clonal Fish, Poecilia formosa
Source: PLoS One. 2016 Aug 2;11(8):e0158442. doi: 10.1371/journal.pone.0158442 (PMC4970819; doi:10.1371/journal.pone.0158442)
Supplement: S2 Table — Here, summary statistics of the 12 microsatellites that were used in differentiating the 7 different clonal lineages of P. formosa are shown, i.e., population, sample size, the number of alleles, the observed heterozygosity of the current generation (H0), the expected heterozygosity (HE), the probability of Hardy-Weinberg-Equilibrium (HWE), i.e., H0 = HE (P), and the FST value of all populations at that particular locus. Note that loci are generally expected not to be in HWE in Amazon mollies, due to the lack of sexual recombination. (PDF) [file pone.0158442.s013.pdf]

S2 Table.

Locus	Characteristics in *Poecilia formosa*							
	Population	*n*	Allele No.	Allele size	H_O	H_E	*P*	F_{ST}
**GA-II41** **Genbank#** AJ810469	Co101	5	2	118 - 126	0.800	0.533	0.429	0.083
	3VI/17	11	2	118 - 126	1.000	0.524	0.003	
	4III/9	12	2	118 - 126	1.000	0.522	0.002	
	W5-Weslaco	7	2	118 - 126	1.000	0.538	0.037	
	6SI-SanIgnacio	5	2	118 - 126	1.000	0.556	0.126	
	7aCS-7aComalSpring	11	2	118 - 126	1.000	0.524	0.003	
	8bCS-8bComalSpring	11	2	118 - 126	1.000	0.524	0.003	
**GA-I47A** **Genbank#** AJ810468	Co101	4	2	133 - 155	1.000	0.571	0.314	0.240
	3VI/17	11	2	133 - 155	1.000	0.524	0.003	
	4III/9	12	2	133 - 155	1.000	0.522	0.002	
	W5-Weslaco	7	2	133 - 159	1.000	0.538	0.037	
	6SI-SanIgnacio	5	2	133 - 155	1.000	0.556	0.128	
	7aCS-7aComalSpring	11	2	133 - 155	1.000	0.524	0.003	
	8bCS-8bComalSpring	11	2	133 - 155	1.000	0.524	0.003	
**GT-II33** **Genbank#** AJ810474	Co101	5	2	178 - 182	0.800	0.533	0.429	0.397
	3VI/17	11	1	182	0.000	0.000	-	
	4III/9	12	1	182	0.000	0.000	-	
	W5-Weslaco	7	1	182	0.000	0.000	-	
	6SI-SanIgnacio	5	1	182	0.000	0.000	-	
	7aCS-7aComalSpring	11	1	182	0.000	0.000	-	
	8bCS-8bComalSpring	11	2	178 - 182	1.000	0.524	0.003	
**GA-V18** **Genbank#** AJ810470	Co101	5	3	122 - 148	1.000	0.644	0.176	0.221
	3VI/17	11	2	122 - 148	1.000	0.524	0.003	
	4III/9	12	2	122 - 148	1.000	0.522	0.002	
	W5-Weslaco	7	2	122 - 148	1.000	0.538	0.037	
	6SI-SanIgnacio	5	2	122 - 144	1.000	0.556	0.127	
	7aCS-7aComalSpring	11	2	122 - 144	1.000	0.524	0.003	
	8bCS-8bComalSpring	11	3	122 - 148	1.000	0.602	0.006	
**GA-I26** **Genbank#** AJ810456	Co101	4	2	160 - 194	1.000	0.571	0.314	0.201
	3VI/17	11	2	160 - 194	1.000	0.524	0.003	
	4III/9	12	2	160 - 194	1.000	0.522	0.002	
	W5-Weslaco	7	2	160 - 194	1.000	0.538	0.038	
	6SI-SanIgnacio	5	2	160 - 194	1.000	0.556	0.126	
	7aCS-7aComalSpring	11	2	160 - 194	1.000	0.524	0.003	
	8bCS-8bComalSpring	11	2	160 - 194	1.000	0.524	0.003	

**GA-III28** **Genbank#** AJ810459	Co101	4	2	215 - 241	1.000	0.571	0.315	0.303
	3VI/17	11	2	215 - 241	1.000	0.524	0.003	
	4III/9	12	2	215 - 241	1.000	0.522	0.002	
	W5-Weslaco	7	2	237 - 241	1.000	0.538	0.038	
	6SI-SanIgnacio	5	2	215 - 241	1.000	0.556	0.126	
	7aCS-7aComalSpring	11	2	215 - 241	1.000	0.524	0.003	
	8bCS-8bComalSpring	11	2	215 - 241	1.000	0.524	0.003	
**GA-III29B** **Genbank#** AJ810460	Co101	5	2	255 - 257	0.000	0.356	0.111	0.889
	3VI/17	11	2	255 - 265	0.000	0.173	0.047	
	4III/9	12	1	255	0.083	0.083	1.000	
	W5-Weslaco	7	1	261	0.000	0.000	-	
	6SI-SanIgnacio	5	1	257	0.000	0.000	-	
	7aCS-7aComalSpring	11	1	257	0.000	0.000	-	
	8bCS-8bComalSpring	11	1	257	0.000	0.000	-	
**GT-I41** **Genbank#** AJ810472	Co101	5	1	148	0.000	0.000	-	-
	3VI/17	11	1	148	0.000	0.000	-	
	4III/9	12	1	148	0.000	0.000	-	
	W5-Weslaco	7	1	148	0.000	0.000	-	
	6SI-SanIgnacio	5	1	148	0.000	0.000	-	
	7aCS-7aComalSpring	11	1	148	0.000	0.000	-	
	8bCS-8bComalSpring	11	1	148	0.000	0.000	-	
**GA-IV42** **Genbank#** AJ810462	Co101	5	3	198 - 204	1.000	0.644	0.175	0.316
	3VI/17	11	2	198 - 202	1.000	0.524	0.003	
	4III/9	12	2	198 - 202	1.000	0.522	0.002	
	W5-Weslaco	7	2	198 - 202	1.000	0.538	0.038	
	6SI-SanIgnacio	5	2	198 - 204	1.000	0.556	0.127	
	7aCS-7aComalSpring	11	2	198 - 204	1.000	0.524	0.003	
	8bCS-8bComalSpring	11	2	198 - 204	1.000	0.524	0.003	
**GA-I29B** **Genbank#** AJ810458	Co101	4	2	229 - 255	1.000	0.571	0.314	0.228
	3VI/17	11	2	229 - 255	1.000	0.524	0.003	
	4III/9	12	2	229 - 255	1.000	0.522	0.002	
	W5-Weslaco	7	2	229 - 257	1.000	0.538	0.037	
	6SI-SanIgnacio	5	2	229 - 255	1.000	0.556	0.127	
	7aCS-7aComalSpring	11	2	229 - 255	1.000	0.524	0.003	
	8bCS-8bComalSpring	11	2	229 - 255	1.000	0.524	0.003	
**GT-II49** **Genbank#** AJ810466	Co101	5	2	342 - 382	1.000	0.556	0.128	0.219
	3VI/17	11	2	342 - 382	1.000	0.524	0.003	
	4III/9	12	2	342 - 382	1.000	0.522	0.002	
	W5-Weslaco	7	2	342 - 382	1.000	0.538	0.037	
	6SI-SanIgnacio	5	2	342 - 382	1.000	0.556	0.126	

	7aCS-7aComalSpring	11	2	342 - 382	1.000	0.524	0.003	
	8bCS-8bComalSpring	11	2	342 - 382	1.000	0.524	0.003	
**GA-III49A NEU**	Co101	5	3	394 - 412	1.000	0.644	0.174	0.181
**Genbank#**	3VI/17	11	2	394 - 408	1.000	0.524	0.003	
AJ810461	4III/9	12	2	394 - 408	0.917	0.518	0.014	
	W5-Weslaco	7	2	394 - 414	1.000	0.538	0.037	
	6SI-SanIgnacio	5	2	394 - 408	1.000	0.556	0.127	
	7aCS-7aComalSpring	11	2	394 - 408	1.000	0.524	0.003	
	8bCS-8bComalSpring	11	2	394 - 408	1.000	0.524	0.003	
