# Supplementary material for: Kin Recognition in a Clonal Fish, Poecilia formosa
Source: PLoS One. 2016 Aug 2;11(8):e0158442. doi: 10.1371/journal.pone.0158442 (PMC4970819; doi:10.1371/journal.pone.0158442)
Supplement: S3 Table — Genetic divergence among the 7 clonal lineages of P. formosa. Above the diagonal are the FST values and below the diagonal are the P-values from the Markov Chain Monte Carlo exact test. Statistical significance after sequential Bonferroni correction for multiple pairwise comparisons is indicated at an experiment-wise error rate α (*α = 0.05; **α = 0.01; *** α = 0.001). (PDF) [file pone.0158442.s014.pdf]

**S3 Table.**

|  | **Co101** | **3VI/17** | **4III/9** | **W5-Weslaco** | **6SI-SanIgnacio** | **7aCS-7aComalSpring** | **8bCS-8bComalSpring** |
| --- | --- | --- | --- | --- | --- | --- | --- |
| **Co101** | - | 0.148** | 0.157** | 0.248** | 0.000 | 0.000 | 0.000 |
| **3VI/17** | 0.00040** | - | 0.000 | 0.251*** | 0.210** | 0.232*** | 0.244*** |
| **4III/9** | 0.00060** | 1.00000 | - | 0.258*** | 0.221** | 0.241*** | 0.253*** |
| **W5-Weslaco** | 0.00085** | <0.00001*** | <0.00001*** | - | 0.298** | 0.318*** | 0.320*** |
| **6SI-SanIgnacio** | 0.00970* | <0.00001*** | <0.00001*** | <0.00001*** | - | 0.000 | 0.000 |
| **7aCS-7aComalSpring** | 0.00045** | <0.00001*** | <0.00001*** | <0.00001*** | 1.00000 | - | 0.006*** |
| **8bCS-8bComalSpring** | 0.39495 | <0.00001*** | <0.00001*** | <0.00001*** | 0.00035** | <0.00001*** | - |
