# Supplementary material for: Kin Recognition in a Clonal Fish, Poecilia formosa
Source: PLoS One. 2016 Aug 2;11(8):e0158442. doi: 10.1371/journal.pone.0158442 (PMC4970819; doi:10.1371/journal.pone.0158442)
Supplement: S4 Table — This table shows the geographical distance (km) between the 7 different clonal lineages above the diagonal. Below the diagonal are the number of loci that are different in their allelic pattern between the different clonal lineages. Interestingly, the San Ignacio clonal lineages is located 552.8 km south of Comal Spring, yet the Comal Spring 7a lineage is identical to the San Ignacio lineage (for the 12 microsatellites that we tested them for) and not to a sympatric clonal lineage Comal Spring 8b. (PDF) [file pone.0158442.s015.pdf]

S4 Table.

	C101	VI/17	III/9	Weslaco	San Ignacio	Comal Spring 7a	Comal Spring 8b
C101	-	654.1	653.9	415.5	573	28.1	28.1
VI/17	6	-	22.51	249.8	100.4	635	635
III/9	6	1	-	255.4	90	634.1	634.1
Weslaco	8	6	6	-	196.2	399.8	399.8
San Ignacio	4	4	4	6	-	552.8	552.8
Comal Spring 7a	4	4	4	6	0	-	0
Comal Spring 8b	3	5	5	7	2	2	-
