# Supplementary material for: Kin Recognition in a Clonal Fish, Poecilia formosa
Source: PLoS One. 2016 Aug 2;11(8):e0158442. doi: 10.1371/journal.pone.0158442 (PMC4970819; doi:10.1371/journal.pone.0158442)
Supplement: S5 Table — The genetic identity within each clonal lineage is higher (i.e., more closely related among each other; 1.000 = 100% genetically identical within clonal lineage) than between clonal lineages, with exception of C101. The lack of higher genetic identity with the C101 clonal lineage may be reflected in the lack of behavioral evidence for kin recognition in this clonal lineage. (PDF) [file pone.0158442.s016.pdf]

S5 Table.

	C101	VI/17	III/9	Weslaco	San Ignacio	Comal Spring 7a	Comal Spring 8b
C101	0.825	0.772	0.772	0.650	0.892	0.892	0.906
VI/17		0.992	0.989	0.750	0.791	0.791	0.750
III/9			0.997	0.708	0.788	0.788	0.747
Weslaco				1.000	0.667	0.667	0.625
San Ignacio					1.000	1.000	0.951
Comal Spring 7a						1.000	0.951
Comal Spring 8b							0.986
