# Supplementary material for: Kin Recognition in a Clonal Fish, Poecilia formosa
Source: PLoS One. 2016 Aug 2;11(8):e0158442. doi: 10.1371/journal.pone.0158442 (PMC4970819; doi:10.1371/journal.pone.0158442)
Supplement: S6 Table — The coefficient of relatedness within each clonal lineage is higher than between clonal lineages, with exception of C101. R = 1: identical twins/clones; R = 0.5: clonal populations as related to each other as full siblings would be in an outcrossing, sexual species; and R<0: less identity than at random (i.e., individuals are as dissimilar to each other as unrelated individuals would be in outcrossing, sexual species with the lower numbers indicating the more unlikely related the lineages are). Note: the underlying logic of R is assuming sexual reproduction of diploid organisms, and therefore, these values are only considered an approximation in clonal organisms. (PDF) [file pone.0158442.s017.pdf]

S6 Table.

	C101	VI/17	III/9	Weslaco	San Ignacio	Comal Spring 7a	Comal Spring 8b
C101	0.115	-0.153	-0.153	-0.77	0.454	0.454	0.525
VI/17		0.960	0.944	-0.264	-0.057	-0.057	-0.264
III/9			0.985	-0.477	-0.072	-0.072	-0.279
Weslaco				1.000	-0.684	-0.684	-0.896
San Ignacio					1.000	1.000	0.752
Comal Spring 7a						1.000	0.752
Comal Spring 8b							0.929
