# Supplementary material for: Kin Recognition in a Clonal Fish, Poecilia formosa
Source: PLoS One. 2016 Aug 2;11(8):e0158442. doi: 10.1371/journal.pone.0158442 (PMC4970819; doi:10.1371/journal.pone.0158442)
Supplement: S7 Table — The strength of preference score (SOP) for several different clonal pairs including: the focal population’s genetic identity; the relatedness coefficient between the focal population and the non-sister stimulus population; the raw preference (s), average SOP and standard deviation for a preference towards the clonal sisters; the raw preference (s), average SOP and standard deviation for a preference towards the non-sister clones; the geographical distance between the two population lineages; and the sample size, t-score, and p-value from the t-tests. Pairs were familiar clonal sister and unfamiliar non-sister, unfamiliar clonal sister and unfamiliar non-sister, or familiar clonal sister and unfamiliar clonal sister. (PDF) [file pone.0158442.s018.pdf]

S7 Table.

	Genetic ID	Relatedness Coefficient	Tested Population	Sister Clones			Non-sister Clone					Statistics		
				Raw SOP	Avg SOP	SD	Raw SOP	Avg SOP	SD	Population	Geo Dist	N	*t*	*P*
familiar sister/ unfamiliar non-sister	0.825	0.454	C101	594.970	0.751	0.096	501.543	0.691	0.097	San Ignacio	573	25	1.567	0.13
	0.986	-0.264	Comal Spring 8b	607.777	0.903	0.154	241.905	0.509	0.188	VI/17	635	18	5.989	< 0.0001
	0.997	-0.279	III/9	657.039	0.852	0.187	330.409	0.566	0.204	Comal Spring 8b	634.1	16	3.484	0.003
	1.000	-0.072	Comal Spring 7a	841.307	0.941	0.152	276.123	0.461	0.205	III/9	634.1	15	5.213	< 0.0001
	1.000	0.752	Comal Spring 7a	823.536	0.946	0.107	244.027	0.463	0.137	Comal Spring 8b	0	10	6.266	<0.0001
	0.992	-0.057	VI/17	767.287	0.914	0.099	272.440	0.505	0.126	Comal Spring 7a	635	15	7.258	< 0.0001
	1.000	1.000	San Ignacio	884.374	0.976	0.040	202.520	0.431	0.052	Comal Spring 7a	552.8	5	13.279	< 0.0001
	1.000	-0.684	Weslaco	682.300	0.886	0.218	287.936	0.518	0.252	San Ignacio	196.2	10	2.491	0.034
	1.000	-0.264	Weslaco	745.248	0.929	0.080	241.126	0.488	0.099	VI/17	249.8	9	7.389	< 0.0001
	1.000	-0.684	Weslaco	820.523	0.980	0.112	192.276	0.418	0.159	Comal Spring 7a	399.8	9	6.225	< 0.0001
unfamiliar sister/ unfamiliar non-sister	1.000	-0.072	Comal Spring 7a	663.391	0.850	0.201	267.998	0.566	0.197	III/9	634.1	15	3.362	0.005
familiar sister/ unfamiliar sister								Unfamiliar Sister clones						
	1.000	1.000	Comal Spring 7a	650.201	0.891	0.199	299.816	0.513	0.255	-	-	14	2.966	0.011
